# Environmental Influence on the Untargeted Foliar Metabolome of Naturally Growing *Mitragyna* Species in Thailand

**DOI:** 10.1002/pei3.70118

**Published:** 2026-01-28

**Authors:** Tushar Andriyas, Nisa Leksungnoen, Chatchai Ngernsaengsaruay, Suwimon Uthairatsamee, Rossarin Tansawat, Pakawat Sirilertpanich

**Affiliations:** ^1^ Department of Forest Biology, Faculty of Forestry Kasetsart University Bangkok Thailand; ^2^ Kasetsart University Research and Development Institute (KURDI), Kasetsart University Bangkok Thailand; ^3^ Center for Advance Studies in Tropical Natural Resources National Research University‐Kasetsart University, Kasetsart University Bangkok Thailand; ^4^ Department of Botany, Faculty of Science Kasetsart University Bangkok Thailand; ^5^ Department of Food and Pharmaceutical Chemistry, Faculty of Pharmaceutical Sciences Chulalongkorn University Bangkok Thailand

**Keywords:** GC–MS, interspecific variations, *Mitragyna* species, secondary metabolite presence, untargeted metabolomics

## Abstract

This study investigates the foliar secondary metabolite profiles of four *Mitragyna* species naturally occurring in Thailand: *M. diversifolia* , *M. hirsuta* , *M. rotundifolia* , and *M. speciosa* (kratom). Using untargeted gas chromatography–mass spectrometry (GC–MS), 409 secondary volatile metabolites were annotated across the four species. *M. diversifolia* exhibited the highest number of detected volatile metabolites (87 ± 7), followed by *M. hirsuta* (75 ± 7), *M. rotundifolia* (74 ± 15), and *M. speciosa* (49 ± 11). Despite its lower overall metabolite count, *M. speciosa* had the highest number of unique compounds distinguishing it from the other species. Ten key volatile metabolites, including mitragynine, speciogynine, speciociliatine, paynantheine, isopaynantheine, and ajmalicine, were identified as major discriminants by Partial Least Squares Discriminant Analysis (PLS‐DA). Leaf traits such as chlorophyll content and leaf pH showed positive correlations with metabolite abundance (*r* = 0.49 and 0.47; *p*‐value < 0.0001), while specific leaf area showed a negative correlation (*r* = −0.51; *p*‐value < 0.0001). Constrained ordination indicated that *T*_max_ (28.04%), vapor pressure deficit, drought, and wind (13.56%) significantly influenced metabolite composition (*p*‐value < 0.001). Given the presence of isomeric volatile metabolites, compound identifications remain putative and will require confirmation through targeted analyses using authenticated standards and orthogonal techniques. These results highlight distinct metabolomic signatures among *Mitragyna* species and provide a foundation for further research and exploration of these species in various scientific and medicinal contexts.

**Abbreviations**
CHL: chlorophyll content
DBH: diameter at breast height
*F*_v_/*F*_m_
: quantum efficiency
LA: leaf area
Lth: leaf thickness
SLA: specific leaf area
ST: stomatal density

## 1 Introduction

*Mitragyna* genus belongs to the Rubiaceae family, classified as a subfamily of Cinchonoideae, tribe Naucleeae, and subtribe Mitragyninae. To date, several *Mitragyna* species have been identified with presence in Africa, including *M. inermis* (Willd.) Kuntze, *M. ledermannii* (K.Krause) Ridsdale, *M. rubrostipulata* (K.Schum.) Havil., and *M. stipulosa* (DC.) Kuntze, and Asia, including *M. diversifolia* (Wall. ex G.Don) Havil., *M. hirsuta* Havil., *M. parvifolia* (Roxb.) Korth., *M. rotundifolia* (Roxb.) Kuntze, *M. speciosa* (Korth.) Havil. or kratom, and *M. tubulosa* (Arn.) Kuntze (Ngernsaengsaruay et al. 2022). *M. diversifolia* , *M. hirsuta* , *M. rotundifolia* , and kratom are the most commonly found *Mitragyna* species in Thailand (León et al. 2009).

The *Mitragyna* species are characterized by deciduous, semi‐deciduous, or evergreen trees growing in tropical forests, swamps, or deserts and savannahs (Razafimandimbison and Bremer 2002), often experiencing periodic rainfall or flooding. To classify the four Thai species, the identification and species characterization of flowers is necessary as the vegetative parts are often misidentified (Ngernsaengsaruay et al. 2022). For instance, the leaf morphology of *M. rotundifolia* and *M. hirsuta* has been reported to be similar, with the species being differentiable only by the shape of calyx of their flowers, while *M. diversifolia* has the smallest leaf among the four *Mitragyna* species (see Figure S1). Further details related to leaf morphology can be found in Figure S1. Kratom has the largest flowering head with a size between 3 and 4 cm compared to a diameter of less than 2.5 cm for the other three species (Ngernsaengsaruay et al. 2022).

Secondary volatile metabolites are differentially distributed across various components of a plant, including the bark, branches, twigs, leaves, fruit, flowers, and roots (Laforest et al. 2023). This differential distribution is suggestive of distinct functional roles related to defense mechanisms in bark and roots, pollinator attraction in flowers, or protection against herbivory and environmental stress in leaves. The production and accumulation of secondary volatile metabolites are usually modulated by biotic and abiotic factors as a survival response to changing environments (Ma et al. 2010; Metlen et al. 2009), which is higher in younger tissue when the plant is more susceptible to environmental stresses (Houghton et al. 1991).

Such responses aid in protection against disease and environmental stresses (Li et al. 2012, 2013), through morphological, anatomical, and physiological adaptation. The accumulation of such chemicals is related to the stage of maturity, geographic origin, ecotype (Boffa et al. 2018), environmental demand, soil type, and soil composition (Chear et al. 2021; Leksungnoen et al. 2022). Variations in specific leaf area, leaf pH, and chlorophyll content can influence the presence and composition of secondary volatile metabolites within plant tissues (Yang et al. 2018). Environmental demands such as temperature fluctuations, air moisture levels, and periodic flooding also impact secondary metabolite synthesis and accumulation patterns.

The presence, biosynthesis, and accumulation of secondary volatile metabolites can vary at the species level due to factors such as differences in genetic makeup, environmental conditions, and interactions with other organisms (Reshi et al. 2023). Studies on various plant species have shown that variations in secondary volatile metabolites can be influenced by genetic differences within and between species, growing locations, processing methods, and plant parts (Li et al. 2022). While secondary volatile metabolites are produced in various *Mitragyna* species as a response to various stressors (Jorge et al. 2016), such metabolic profiles have not been previously characterized under natural, contiguous growing conditions.

Metabolomics accompanied with analytical techniques, including metabolite profiling through untargeted analysis can shed light on the presence, accumulation and biological pathways of secondary volatile metabolites. These include gas chromatography–mass spectrometry (GC–MS), liquid chromatography–mass spectrometry (LC–MS), and nuclear magnetic resonance (NMR) (Benkeblia 2023). GC–MS has several advantages over liquid‐phase techniques like LC–MS as it can be used to analyze volatile and thermally stable compounds, providing high‐resolution separation and reproducible retention times due to the robustness of gas chromatography (Sparkman et al. 2011). Moreover, GC–MS is relatively less expensive and needs less complex sample preparation compared to LC–MS. However, its reliance on derivatization for non‐volatile and thermally labile volatile metabolites introduces complexity and potential variability (Dunn and Ellis 2005; Fiehn 2002). Furthermore, the high maintenance and calibration demands of GC–MS systems, particularly for ensuring reproducible results, add to the operational challenges. While these challenges restrict its application in comprehensive metabolomic studies, GC–MS remains a valuable tool in complex biological systems.

There has been a long standing interest in the *Mitragyna* genus since the early 19th century due to a rich secondary metabolite profile, with antioxidant, antimicrobial, anticancerous, and enzyme‐inhibitory activities (Shunmuga Jothi et al. 2015). These include alkaloids from the cinchona and yohimbine groups, such as mitragynine and mitraversine (Barger et al. 1939). Other reported compounds include polyphenols, flavonoids, triterpenoids, triterpenoid saponins, monoterpenes, and secoiridoids (Brown et al. 2017). Hence, the species find use in traditional ethno‐medicines for treating a variety of maladies (Brown et al. 2017; Taneja et al. 2023).

To date, only a few studies have reported the metabolomic profile of *Mitragyna* species growing naturally in various climatic and soil conditions, with significant focus given to kratom due to its pharmacologically active alkaloids. The objective of this study was to determine the secondary metabolite profile of the four *Mitragyna* species growing in various parts of Thailand under varying environmental conditions and to compare the interspecific differences in their metabolite profiles. Additionally, we also analyzed the significant abiotic factors affecting the variability of the number of secondary metabolites identified, using a constrained ordination analysis. We were interested in determining whether morphologically similar *Mitragyna* species ( *M. hirsuta* and *M. rotundifolia* ; see Figure S1), determined through a previous study (Ngernsaengsaruay et al. 2022), can be reliably differentiated based on their foliar metabolomic profiles using untargeted analysis of leaves collected from co‐occurring trees in multiple regions across Thailand.

## 2 Methods

### 2.1 Tree Sampling and Physiological Measurements

**FIGURE 1:**
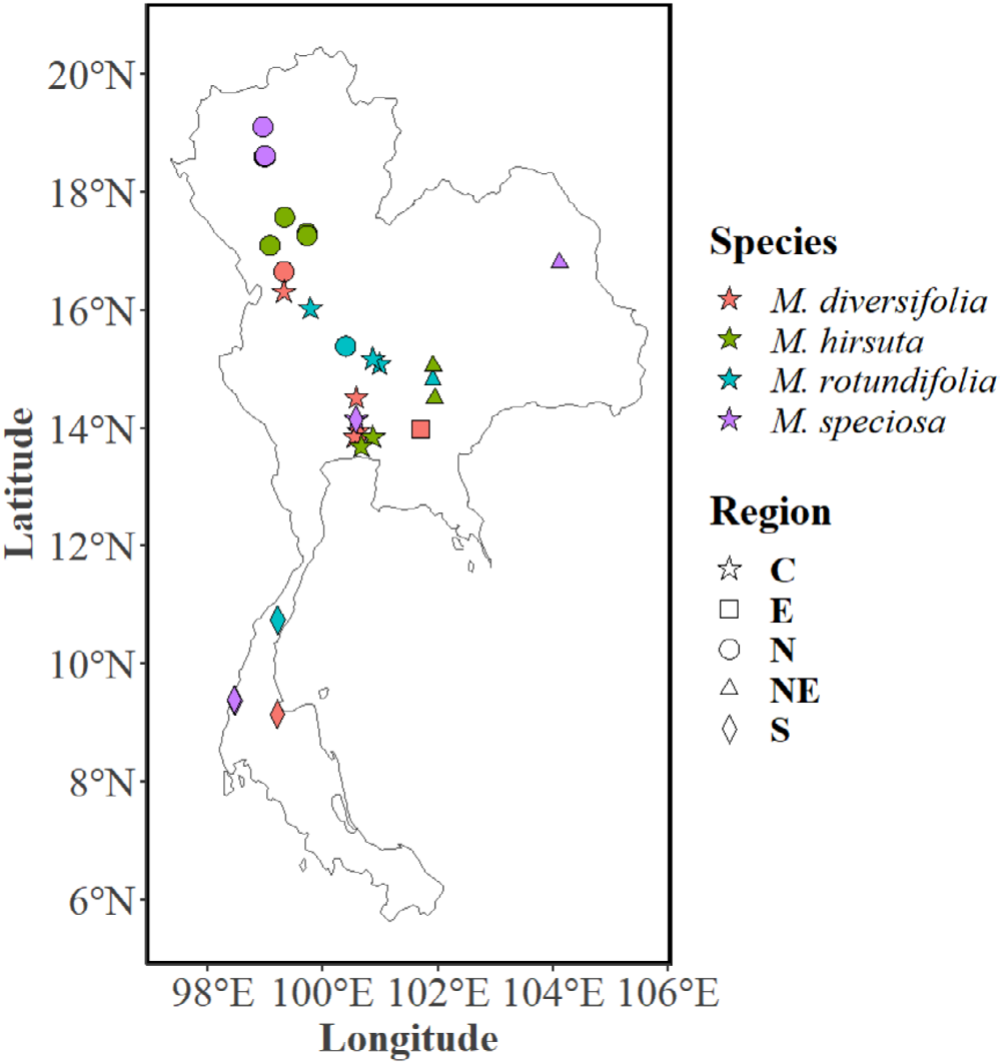
Distribution of the *Mitragyna* species growing in various parts of Thailand. The four species are *Mitragyna diversifolia* (Wall. ex G.Don) Havil., *M. hirsuta* Havil., *M. rotundifolia* (Roxb.) Kuntze, and *M. speciosa* (Korth.) Havil. or kratom.

Sixty three individual trees of the four *Mitragyna* species ( *M. diversifolia* , *M. hirsuta* , *M. rotundifolia* , and kratom) were sampled from various parts of Thailand and identified by a taxonomist from the Department of Botany, Faculty of Science, Kasetsart University (Ngernsaengsaruay et al. 2022). The sample specimens, location and elevation, and tree characteristics such as stem size (girth at breast height: GBH), height, and canopy area (Table S1) were measured. The trees were randomly sampled from various parts of Thailand experiencing different climatic conditions (Figure 1), which included locations from the North (N), Central (C), East (E), Northeast (NE), and South (S), as indicated in Table S1. Additionally, leaf functional traits were measured for each tree as outlined in the previous paper (Leksungnoen et al. 2022). Soil samples were also collected from these sites and are enumerated in the previous work (Leksungnoen et al. 2022).

Physiological leaf sampling was conducted using sun‐exposed, fully developed leaves selected from the second or third node below the shoot apex to ensure physiological maturity. Samples were taken from the outer portion of the canopy, with the canopy center used as a positional reference. Leaves that were completely exposed to direct sunlight or exhibited visible symptoms of disease or insect damage were excluded from sampling. For each tree, 10 leaves were collected and used for trait measurements.

Physiological leaf traits related to growth in individual trees (63 trees) including leaf area (LA), specific leaf area (SLA), leaf thickness (Lth), chlorophyll content (CHL), maximum quantum efficiency (*F*_v_/*F*_m_), performance index (PI), stomatal density (ST), and leaf pH (pH) were measured. In each tree, 10 expanded and mature second or third leaves were collected from the shoot to avoid any age variability in the sampled leaves. Henceforth, LA was measured using an image processing software (ImageJ, U.S. National Institutes of Health, Bethesda, Maryland, USA, https://imagej.nih.gov/ij). Given that SLA is calculated as the ratio of LA and the dry leaf mass, the leaf samples used to measure LA were oven‐dried at 60°C for 48 h. and their dry mass was measured using a 4‐digit analytical balance (AE200 Mettler Toledo LLC, Columbus, Ohio, USA). Leaf thickness was measured on the lamina by avoiding the vein using a digital leaf thickness meter (Digimatic Thickness Guage, Model 547 Mitutoyo Cooperation, Japan).

Chlorophyll content or CHL was measured using a SPAD meter (Model SPAD‐502, Konica Minolta Inc., Osaka, Japan). Each leaf was measured at five different locations and their average was reported in units of SPAD. *F*_v_/*F*_m_ and PI were measured using a chlorophyll fluorometer (Model OS‐30p+, Opti‐Sciences Inc., Hudson, NH, USA). Two sliding clips were attached to each chosen leaf and the setup was left for 15–30 min for the leaf to adapt to the ambient dark lighting conditions before the measurement. Subsequently, the measured values were averaged to obtain a value per leaf.

ST was measured using the nail‐polish imprint method (Yin et al. 2020). A clear nail polish was applied onto three locations (base, middle, and apex) on lower side of the leaf where most of the stomata are found. After the nail polish dried up, a sellotape was pressed onto the leaf and then pulled off and mounted onto a glass slide. Pictures were taken under a digital light microscope having a zoom between 10× and 40×. The total number of stomata were counted in an area of 1 mm^2^ using the ImageJ software. For the leaf pH, 5 g of fresh leaf was ground with 40 g of water (in a ratio of 1:8) and the mixture was shaken and left for 30 min. The mixture was filtered with a filter paper (No. 1 Whatman, Sigma‐Aldrich Pte Ltd., Singapore) and the pH was measured using a pH meter (AMTO1, AMTAST USA Inc., Florida, USA).

### 2.2 TerraClimate Data

Climate data was obtained from the TerraClimate global climate dataset (Abatzoglou et al. 2018), publicly available at https://climate.northwestknowledge.net/TERRACLIMATE. TerraClimate is a global dataset with a spatial resolution of 0.04° (approximately 4 km at the equator) and monthly temporal resolution, available from the year 1958 to 2020 and is climatically interpolated from monthly station data obtained from the WorldClim dataset (Abatzoglou et al. 2018). The dataset includes several climate and environmental variables (primary and secondary), including air temperature extremes (minimum/maximum), precipitation, downward shortwave surface radiation (SRAD), vapor pressure (VP), vapor pressure deficit (VPD), wind speed, climatic water deficit, soil water equivalent, runoff, soil moisture, Palmer Drought Severity Index (PDSI, which indicates the drought severity based on precipitation and temperature data), theoretical reference (potential) precipitation based on computations, and measured evapotranspiration based on remote sensing data. These data were obtained from the public domain and subsequently extracted and analyzed using R statistical software (R Core Team 2023).

### 2.3 Analysis of Secondary Metabolites Using Gas Chromatography‐Mass Spectrophotometer

The extract was prepared as described in (Leksungnoen et al. 2022). Briefly, the leaf samples were thoroughly cleaned to remove any dirt particles and were dried in a hot air oven at 45°C until they reached a constant mass. Twenty grams of dried ground leaves were then soaked in 200 mL of methanol (MeOH) for 3 days at room temperature and shaken occasionally. Briefly, oven‐dried leaf samples were ground into a fine powder and macerated in methanol (99%, v/v) at a ratio of 1:10 (w/v) for 3 days at room temperature. During the extraction period, the solvent was replenished daily with fresh methanol, and the mixture was occasionally shaken to facilitate solvent penetration and improve extraction efficiency. The mixture was then filtered with Whatman No. 1 (Merck Ltd., Darmstadt, Germany) and concentrated using a rotary vacuum evaporator (Buchi, Rotavapor R‐100, Switzerland) under reduced pressure at 40°C. Approximately 10 mg of this extract was then dissolved in 50% MeOH (1 mL), sonicated for 30 min, and centrifuged for 5 min at 12,000 rpm.

The supernatant was then subjected to gas chromatography–mass spectrometry analysis using QP2020 NX (Shimadzu Co., Japan) equipped with SH‐Rxi‐5Sil MS column (0.25 μm df × 0.25 mm ID × 30 m length). Helium (99.9%) was used as the carrier gas at a flow rate of 1 mL/min. Samples were injected at a volume of 0.7 μL using the splitless mode at a sampling time of 1 min. The ion source and interface temperatures of the mass spectrometer as well as the injector temperature were maintained at 250°C. The mass spectra were obtained through electron ionization (EI) at 70 eV, using a mass scan range between *m/z* 45–700, at a scan speed of 2500 and an event time of 0.3 s, respectively. The column oven temperature program was initiated at 60°C (held for 3 min), which was then raised at a rate of 8°C/min to 280°C (held for 25 min).

The raw chromatograms and mass spectra obtained from gas chromatography–mass spectrometry were processed to detect peak heights significantly above the noise floor. After preprocessing through baseline correction, peak alignment, and normalization, statistical analysis was used on peak intensities normalized across samples to compare with databases through mass spectral data and retention times. The compounds that were annotated were those within mass spectra which were at least 80% similar to that in the NIST database (https://webbook.nist.gov/chemistry/) as well as publicly available databases for mass spectrometry, including MassBank (http://www.massbank.jp) and METLIN (http://metlin.scripps.edu/index.php). An 80% similarity threshold for compound identification is commonly used in metabolomic studies to balance specificity and sensitivity in compound identification (Stein 1999; Zhu et al. 2023). While higher thresholds (greater than 90%) are preferable (Kind and Fiehn 2007), they often exclude low‐abundance compounds or those with incomplete reference spectra (Summer et al. 2007).

### 2.4 Statistical Analysis

One‐way ANOVA was used to compare the mean growth and leaf traits among the four regions of Thailand (Table 1) using the R statistical software (R Core Team 2023). Post hoc mean comparisons were tested using Tukey's honestly significant difference (HSD) test using the R package *agricolae* (Mendiburu 2021). Canonical correspondence analysis (CCA) was used to determine the variations in the number of chemical compounds in the four *Mitragyna* species constrained by the environmental and soil predictors. In this direct gradient technique, sites, environmental variables, and chemicals are simultaneously represented in a lower dimensional space (Ter Braak 1987). The constrained CCA axes represent a linear combination of environmental variables responsible for the maximum separation or variation between the sampled locations. The eigenvalue of each CCA axis indicates its relative importance and is a measure of the extent to which a linear combination of environmental variables can explain the variations in the number of compounds (Van Tongeren et al. 1995).

**TABLE 1:** Leaf traits of the four *Mitragyna* species growing naturally in Thailand.

Leaf traits	*M. diversifolia* (MD)	*M. hirsuta* (MH)	*M. rotundifolia* (MR)	*M. speciosa* (MS)	*p*
Leaf area (LA) (m^2^)	29.52 ± 7.15c	152.11 ± 51.69ab	219.00 ± 113.11a	106.8 ± 21.85b	< 0.001***
Specific leaf area (SLA) (cm^2^g^−1^)	134.51 ± 37.94a	102.96 ± 30.57b	126.45 ± 40.58ab	155.14 ± 22.39a	< 0.001***
Leaf thickness (Lth) (mm)	0.24 ± 0.06a	0.27 ± 0.06a	0.24 ± 0.03a	0.17 ± 0.01b	< 0.001***
Chlorophyll content (CHL) (SPAD)	29.43 ± 3.79b	34.09 ± 3.53a	31.70 ± 4.70ab	33.12 ± 3.08a	0.004*
Quantum yield (*F*_v_/*F*_m_) (unitless)	0.80 ± 0.03	0.79 ± 0.02	0.80 ± 0.01	0.78 ± 0.04	0.400
Performance index (PI) (unitless)	2.73 ± 1.20	2.59 ± 0.68	2.58 ± 0.58	2.39 ± 0.90	0.791
Stomatal density (ST) (No. mm^−2^)	253.14 ± 44.78b	261.47 ± 53.55b	257.51 ± 71.66b	331.93 ± 50.11a	< 0.001***
Leaf pH (pH) (unitless)	4.79 ± 0.22b	5.09 ± 0.38a	5.00 ± 0.25ab	4.48 ± 0.25c	< 0.001***

*Note:* Lowercase letters within each row represent the results of one‐way analysis of variance (ANOVA) followed by Tukey's honestly significant difference (HSD) test. Means sharing the same letter are not significantly different at the 95% confidence level. Statistical significance is indicated as * for *p*‐value < 0.05, *** for *p*‐value < 0.01, and ** for *p*‐value < 0.001.

A forward selection using Monte Carlo testing with 999 unrestricted permutations was used to identify the most significant environmental variables (*p*‐value < 0.01). The environmental variables with a significant effect on the number of compounds were then illustrated as a triplot (i.e., the environmental variables, leaf traits, number of compounds, and the sample locations in the four regions of Thailand). The analysis was undertaken using the CCA functions available in the *vegan* package (Dixon 2003; Oksanen et al. 2007) P of the R statistical language (R Core Team 2023).

The metabolite profiles of leaves from four *Mitragyna* species were analyzed using Principal Component Analysis (PCA), Partial Least Squares Discriminant Analysis (PLS‐DA), volcano plots, and boxplots. Data normalization via log transformation and Pareto scaling was followed by fold change analysis (|log_2_fc| > 2) and *t*‐tests with FDR‐adjusted *p*‐value below 0.05 (Benjamini‐Hochberg correction as in Benjamini and Hochberg (1995)). Multivariate analyses, including unsupervised PCA and supervised PLS‐DA, were performed to identify key metabolites differentiating the species based on scaled Variable Importance in Projection (VIP) scores. To ensure model accuracy, the dataset was split into training and testing sets (at a ratio of 75:25) through k‐fold cross‐validation. Boxplots and heatmaps facilitated visualization of interspecific metabolic variations.

## 3 Results

### 3.1 Leaf Functional Traits

Among the four *Mitragyna* species, *M. rotundifolia* had the largest leaf (219.00 ± 113.11 cm^2^) with a high variance as indicated by the LA (Table 1) followed by *M. hirsuta* and kratom, with *M. diversifolia* (29.52 ± 7.15 cm^2^) having the smallest leaf. SLA, which is the ratio of the LA and the leaf dry mass, was the highest for kratom and *M. diversifolia* , while *M. hirsuta* had the lowest SLA. SLA exhibited a significant negative correlation with the number of secondary volatile metabolites (*r* = −0.508, *p*‐value < 0.0001), while CHL content showed a positive association with metabolite presence (Figure S2). The location‐wise leaf functional traits of *Mitragyna* species are listed in Table S2. Kratom had the thinnest leaf, while the remaining three species had a similar thickness. CHL, *F*_v_/*F*_m_, and PI are directly related to the light reaction processes during photosynthesis. Within the four *Mitragyna* species, *M. diversifolia* had the lowest CHL, while *F*_v_/*F*_m_ and PI were not statistically different (*p*‐value > 0.05), indicating that for a given light intensity, *M. diversifolia* would have a relatively slower growth rate.

Kratom had the highest ST relative to the other three species. ST is related to the gas exchange during the Calvin cycle along the photosynthetic pathway, with a higher density indicating a higher uptake of CO_2_. All four species had an acidic leaf pH (< 7), with kratom having the lowest leaf pH (more acidic), followed by *M. diversifolia* , *M. rotundifolia* , and *M. hirsuta* . Furthermore, leaf pH correlated positively with secondary metabolite abundance (*r* = 0.470, *p*‐value < 0.0001), indicating that less acidic leaf conditions were linked to a higher presence of secondary volatile metabolites (Figure S2). In short, as indicated by leaf traits such as high SLA, CHL, and ST, kratom would have a higher growth rate compared to the other *Mitragyna* species, with *M. diversifolia* having the slowest growth rate (smallest LA, low SLA, lowest CHL, and lowest ST).

### 3.2 Canonical Correspondence Analysis

**FIGURE 2:**
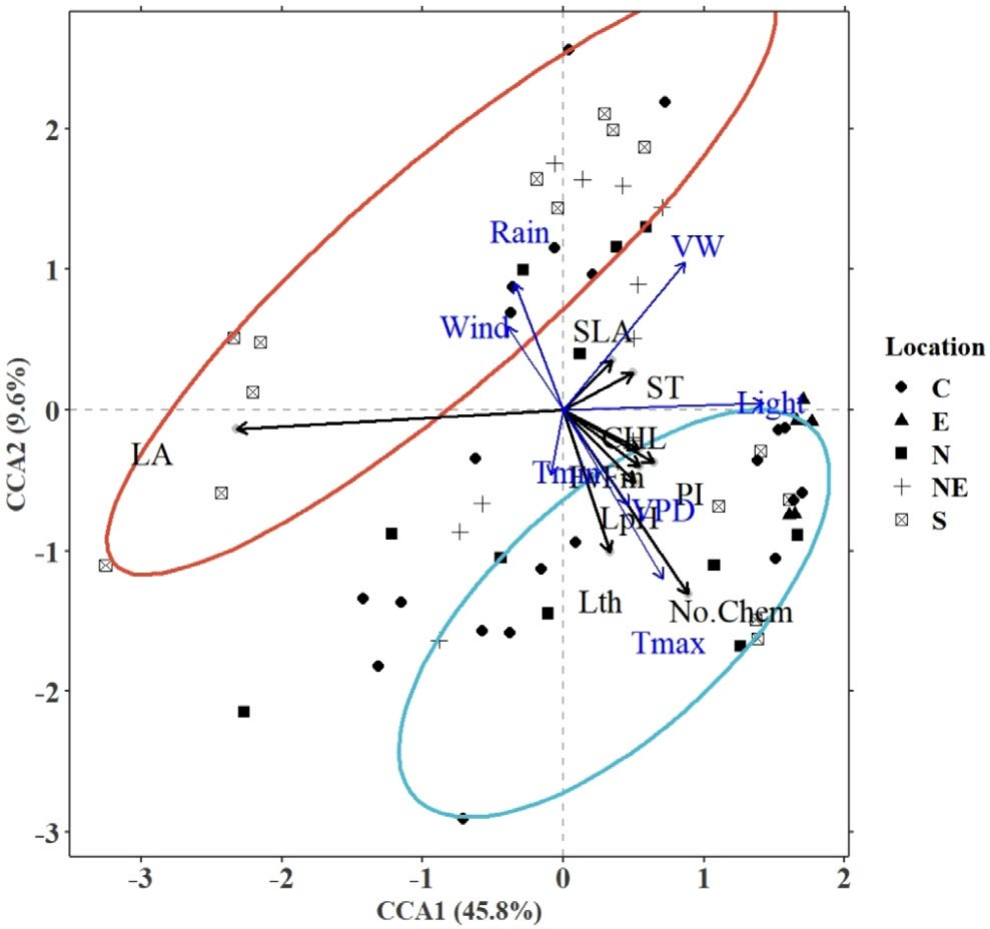
CCA ordination plot for the significant (*p*‐value < 0.05) leaf traits and mean environmental variables in the mean sub‐model influencing the chemical abundance in the sampled trees from various locations (C: Central Thailand, E: Eastern Thailand, N: Northern Thailand, NE: Northeast Thailand, and S: Southern Thailand). The ellipses represent 95% confidence levels for number of chemicals (blue for high and red for low). The leaf traits analyzed were leaf area (LA), specific leaf area (SLA), leaf thickness (Lth), chlorophyll content (CHL), quantum efficiency (*F*_v_/*F*_m_), performance index (PI), stomatal density (ST), and leaf pH (pH).

**FIGURE 3:**
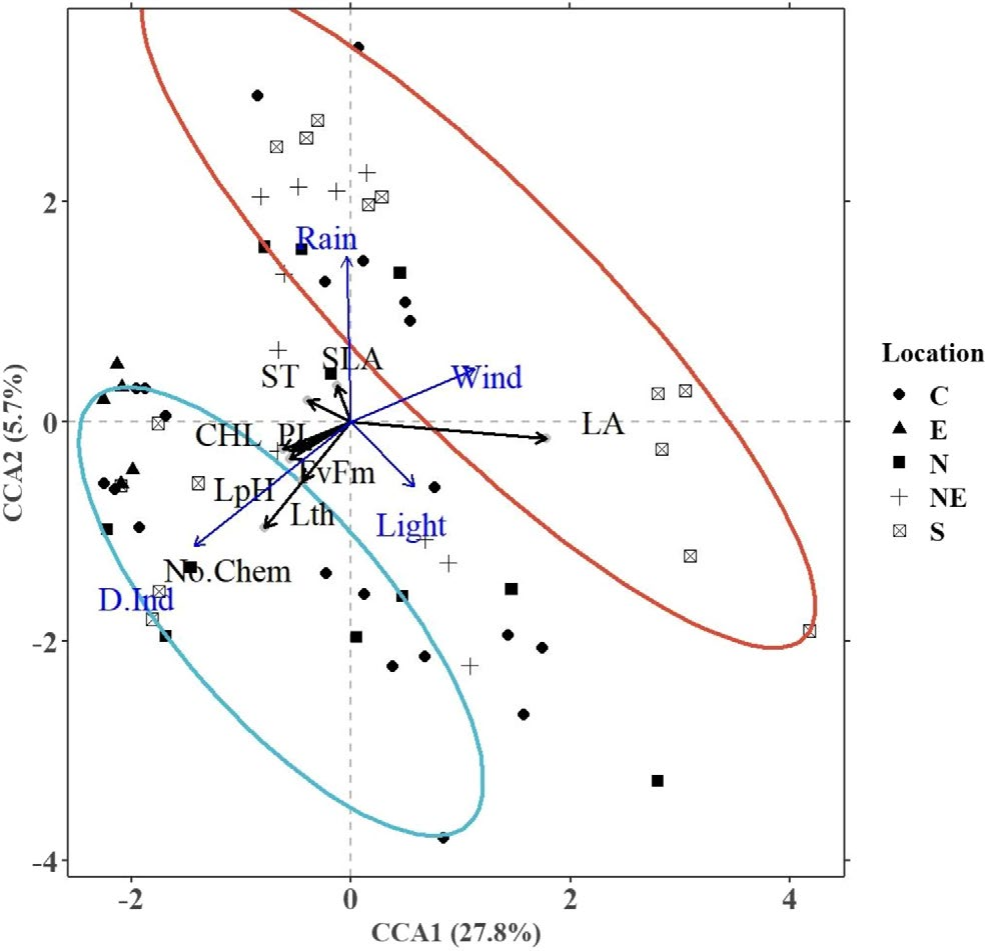
CCA ordination plot for the leaf traits and standard deviations of the environmental variables found significant (*p*‐value < 0.05) in the SD or standard deviation sub‐model influencing the chemical abundance in the sampled trees from various locations (C: Central Thailand, E: Eastern Thailand, N: Northern Thailand, NE: Northeast Thailand, and S: Southern Thailand). The ellipses represent 95% confidence levels for number of chemicals (blue for high and red for low). The leaf traits analyzed were leaf area (LA), specific leaf area (SLA), leaf thickness (Lth), chlorophyll content (CHL), quantum efficiency (*F*_v_/*F*_m_), performance index (PI), stomatal density (ST), and leaf pH (pH).

We used CCA to determine the most significant environmental and soil variables influencing the leaf traits and the total number of chemicals in the four *Mitragyna* species at various locations in Thailand. Two CCA sub‐models were built, one constrained with only the mean climate and soil variables, and the other with only the standard deviation of the climate variables, as calculated using a 30‐year climate dataset. The results are plotted in Figure 2 (mean sub‐model) and Figure 3 (standard deviation sub‐model) and quantified in Table 2. Both the sub‐models were found to be highly significant (*p*‐value < 0.001). Collinear variables were removed by using the variance inflation factor or *vif* function in R statistical software and a subsequent forward selection indicated that *T*_max_, *T*_min_, VPD, wind, light, rain, and VW in the mean sub‐model and wind, light, rain, and PDSI in the standard deviation sub‐model were highly significant. It was observed that *T*_max_ (mean sub‐model) and wind (standard deviation sub‐model) explained most of the total variance (around 28% and 14%, respectively) in the respective sub‐models.

**TABLE 2:** Canonical correspondence analysis (CCA) of environmental variables (sub‐model (1) mean climate data and soil properties sub‐model (2) standard deviation of climate data). The results indicate the significance of variations in leaf traits and number of chemicals through the significant explanatory variables (the remaining variables were removed through forward selection analysis), using Monte Carlo permutation test (999 permutations) at the significance level of 99%.

Environmental variable	Marginal effect (%; *p*)	Conditional effect (%; *p*) [forward selection]	Pure effect (%; *p*)
Mean sub‐model			
1. *T*_max_ (°C)	9.30 (0.007**)	16.16 (0.001***)	28.04 (0.001***)
2. *T*_min_ (°C)	1.21 (0.477^ns^)	4.91 (0.002***)	1.64 (0.235^ns^)
3. VPD (kPa)	3.76 (0.086°)	5.02 (0.002***)	4.69 (0.007**)
4. Wind (m s^−1^)	2.68 (0.162^ns^)	4.69 (0.004**)	2.97 (0.025*)
5. Light (MJ m^−2^ day^−1^)	23.26 (0.001***)	11.49 (0.001***)	10.31 (0.001***)
6. Rainfall (mm)	3.40 (0.112°)	4.24 (0.003**)	2.36 (0.002**)
7. Palmer DI (unitless)	6.09 (0.031*)	1.55 (0.131^ns^)	—
8. Volumetric soil moisture (VW) (%)	11.59 (0.005**)	7.50 (0.002**)	7.72 (0.001***)
9. Bulk density (g cm^−3^)	7.16 (0.026*)	0.20 (0.853^ns^)	—
10. Soil pH (unitless)	9.81 (0.002**)	1.53 (0.116^ns^)	—
11. Organic matter (OM) (%)	5.31 (0.052°)	1.10 (0.245^ns^)	—
12. Carbon (C) (%)	2.66 (0.226°)	2.49 (0.037*)	—
13. Nitrogen (N) (%)	5.06 (0.054°)	2.33 (0.037*)	—
14. Phosphorus (P) (mg kg^−1^)	0.35 (0.87^ns^)	0.45 (0.615^ns^)	—
15. Potassium (K) (mg kg^−1^)	5.56 (0.039*)	0.70 (0.441^ns^)	—
16. Calcium (Ca) (mg kg^−1^)	2.23 (0.26^ns^)	0.44 (0.658^ns^)	—
17. Magnesium (Mg) (mg kg^−1^)	1.10 (0.481^ns^)	0.83 (0.341^ns^)	—
Standard deviation sub‐model			
1. *T*_max_ (°C)	2.96 (0.141^ns^)	3.24 (0.051°)	—
2. *T*_min_ (°C)	3.61 (0.096°)	0.94 (0.501^ns^)	—
3. VPD (kPa)	3.84 (0.101^ns^)	2.23 (0.151^ns^)	—
4. Wind (m s^−1^)	9.42 (0.008**)	8.13 (0.001***)	13.56 (0.001***)
5. Light (MJ m^−2^ day^−1^)	5.06 (0.05*)	5.75 (0.004**)	3.85 (0.009**)
6. Rainfall (mm)	3.37 (0.137^ns^)	9.21 (0.002**)	12.70 (0.001***)
7. Palmer DI (unitless)	16.43 (0.001***)	6.79 (0.002**)	6.06 (0.001***)

*Note:* Highlighted rows indicate variables selected through forward elimination and used to constrain the CCA (ns: not significant). Environmental variables (mostly with first constrained axis for Temp and light). VW first, P with second, and Ca with both constrained axis ‘***’ 0.001, ‘**’ 0.01, ‘*’ 0.05, ‘^o^’ 0.1, (ns: not significant).

#### 3.2.1 Mean Sub‐Model

The first two CCA axes of this sub‐model (constrained with VPD, wind, light, rain, and VW) explained over 55% of the variance in the data. As seen in Figure 2, *T*_max_, *T*_min_, and VPD induced the production of a higher number of chemicals. However, rainfall and wind reduced the number of SMs, with a higher mean rainfall and wind speed resulting in a lower number of chemicals. Additionally, leaf traits positively related to high growth, including Lth, pH, PI, *F*_v_/*F*_m_, and CHL, were found to be conducive to the production of a higher number of chemicals.

#### 3.2.2 Standard Deviation Sub‐Model

The CCA axes of this sub‐model (constrained using wind, light, rain, and PDSI) explained over 33% of the variance in the data (Figure 3). Deviations in wind (13.56%) had the strongest influence in the sub‐model and resulted in a reduced number of chemicals in the profile, while deviations in PDSI significantly influenced a higher number of chemicals. Although deviations in rainfall amount did not have a significant marginal effect (i.e., variance explained by a model with rainfall as the only constraining variable), they did have significant conditional and pure (i.e., variance explained by the variable with the remaining significant variables used as co‐variates) effects.

### 3.3 Statistical Analysis of Secondary Volatile Metabolites

**FIGURE 4:**
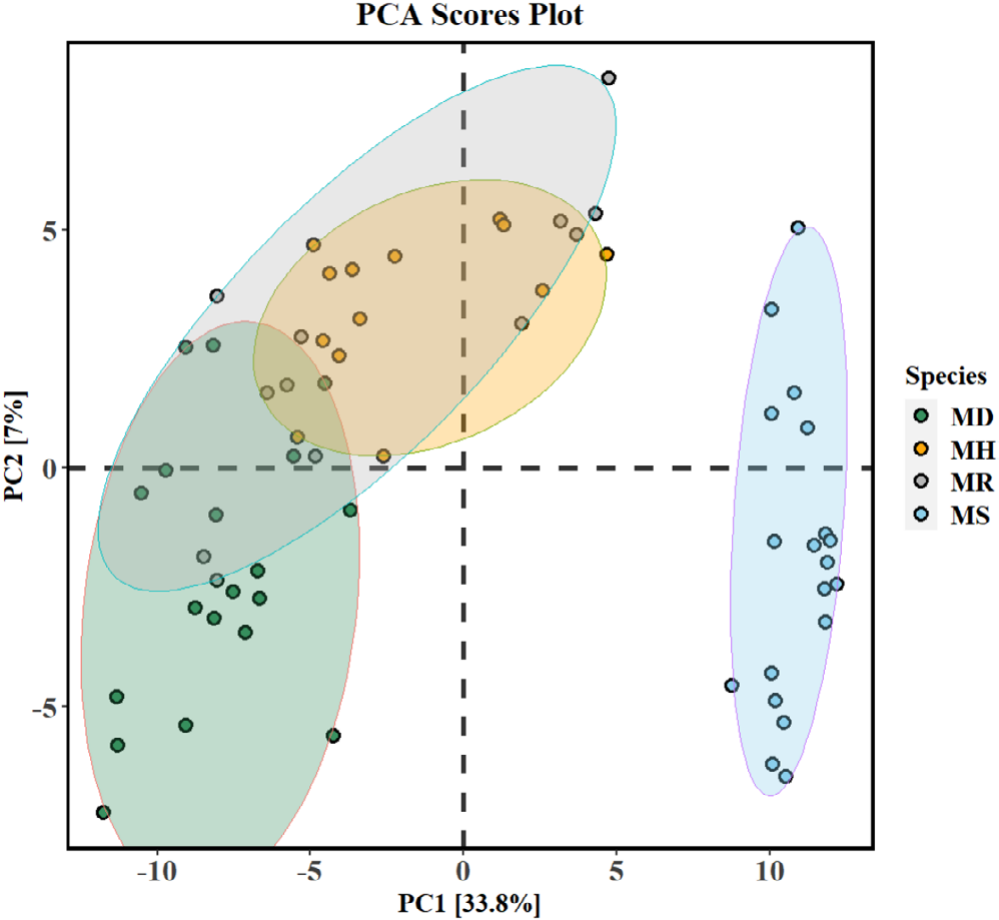
Exploratory principal component analysis (PCA) scores plot of the metabolomic distribution in the leaves of the four *Mitragyna* species (shaded ellipses representing 95% confidence intervals) and the variance explained. The four species are *Mitragyna diversifolia* (Wall. ex G.Don) Havil. or MD, *Mitragyna hirsuta* Havil. or MH, *Mitragyna rotundifolia* (Roxb.) Kuntze or MR, and *Mitragyna speciosa* (Korth.) Havil. or MS.

Using gas chromatography–mass spectrometry on MeOH extract, 409 secondary volatile metabolites were identified in the four *Mitragyna* species and are listed in the Supporting Information, Excel Table S1. Representative chromatograms and mass spectra of two sampled trees of *M. speciosa* and *M. diversifolia* can be found in the Supporting Information *Kratom_1.2_10mgml.pdf* and *MD1.pdf*, respectively. We defined the presence as the average number of secondary volatile metabolites per species, with *M. diversifolia* having the highest presence of distinct chemicals (87 ± 7), followed closely by *M. rotundifolia* and *M. hirsuta* with similar numbers (74 ± 15 and 75 ± 7, respectively), whereas kratom had the lowest number of secondary volatile metabolites (49 ± 11). Through a multivariate analysis, the differences in metabolite composition between the leaves of the four species were determined. The unsupervised PCA scores plot (Figure 4) indicated that kratom formed a distinct cluster, while the clusters of samples from *M. diversifolia* , *M. rotundifolia* , and *M. hirsuta* were not well separated (samples were clustered under shaded ellipses representing 95% confidence intervals). The model variance explained by the first two principal axes was above 40% and within the 409 volatile metabolites, 45 were found to significantly influence the loadings along PC1 and PC2 axes (Supporting Information, Excel Table S2).

**FIGURE 5:**
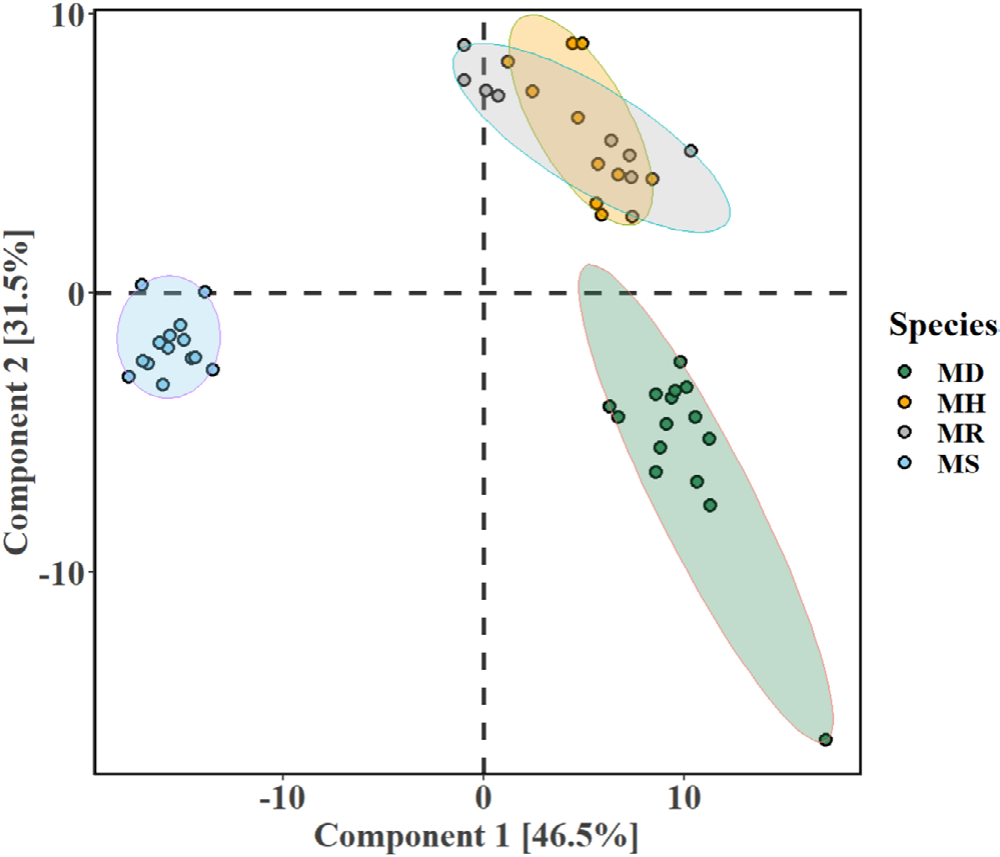
Partial least squares discriminant analysis (PLS‐DA) of metabolomics data for the four *Mitragyna* species. The four species are *Mitragyna diversifolia* (Wall. ex G.Don) Havil. or MD, *Mitragyna hirsuta* Havil. or MH, *Mitragyna rotundifolia* (Roxb.) Kuntze or MR, and *Mitragyna speciosa* (Korth.) Havil. or MS.

A supervised PLS‐DA model was used to identify metabolites that were significant in classifying the four species and revealed distinct clustering patterns. The data was equally split into training and testing sets with the PLS‐DA scores plot demonstrating a clear separation between the metabolomes of MS, *M. diversifolia* , while the *M. hirsuta* and *M. rotundifolia* samples had a distinct overlap (Figure 5). The classification accuracy as obtained from the testing set was around 86%. Scaled VIP scores greater than 1 were used to identify metabolites that best explained the discrimination between metabolomes of the four species.

**FIGURE 6:**
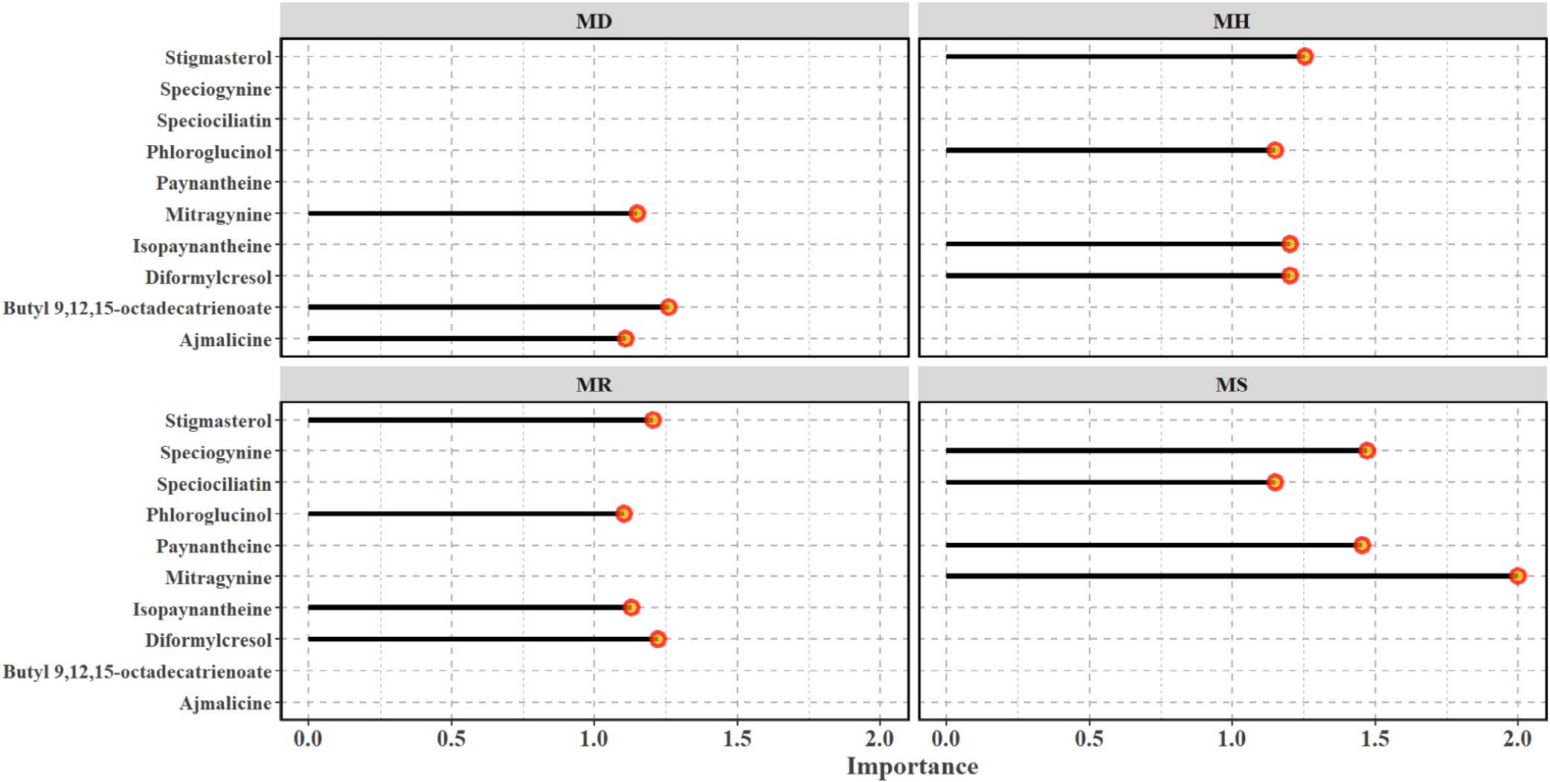
Scaled variable importance in projection (VIP) scores for the volatile metabolites most important in discriminating between the four *Mitragyna* species as per the PLS‐DA. The four species are *Mitragyna diversifolia* (Wall. ex G.Don) Havil. or MD, *Mitragyna hirsuta* Havil. or MH, *Mitragyna rotundifolia* (Roxb.) Kuntze or MR, and *Mitragyna speciosa* (Korth.) Havil. or MS.

Among the 409 volatile metabolites, a total of 10 volatile metabolites were identified as significant discriminants, including ajmalicine; butyl 9,12,15‐octadecatrienoate; mitragynine; phloroglucinol; diformylcresol; isopaynantheine; stigmasterol; speciogynine; speciociliatin; and paynantheine (Figure 6). A closer look at the volatile metabolites that were important in classifying the groups in PLS‐DA indicated that paynantheine and mitragynine positively loaded PC2 (figure in Supporting Information) and were present abundantly in kratom (see Figure 6), while butyl 9,12,15‐octadecatrienoate, which had a high presence in *M. diversifolia* , negatively loaded PC1. The presence of stigmasterol, which equally loaded positive PC1 and negative PC2, was elevated in kratom relative to other species. This was in contrast to speciogynine, which had a higher presence in kratom, and equally loaded PC1 (negative loading) and PC2 (positive loading).

**FIGURE 7:**
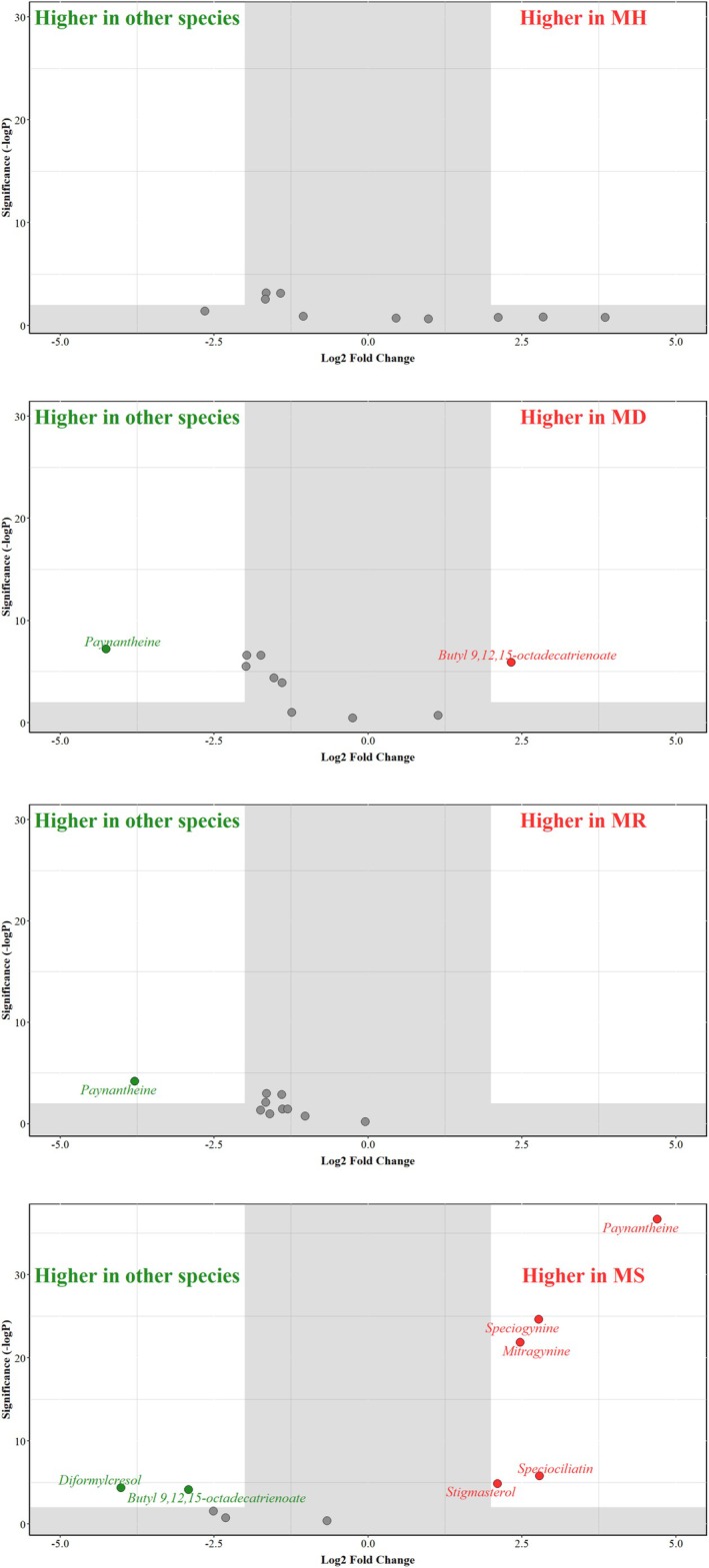
Scatter volcano plots depicting the fold change (FC) and *t*‐test *p*‐value of the identified significant volatile metabolites in the four species. The four species are *Mitragyna diversifolia* (Wall. ex G.Don) Havil. or MD, *Mitragyna hirsuta* Havil. or MH, *Mitragyna rotundifolia* (Roxb.) Kuntze or MR, and *Mitragyna speciosa* (Korth.) Havil. or MS. The *X*‐axis represents the log_2_‐transformed FC, while the *Y*‐axis represents the log_10_‐transformed *p*‐value. The cutoff region (depicted in shaded gray rectangles) was defined by a log_2_ fold change > 4 and −log FDR adjusted *p*‐value above 2. The circles represent ten volatile metabolites identified as significant interspecific discriminators through the PLS‐DA. The red circles indicate volatile metabolites with a higher presence while those in green indicate volatile metabolites with lower presence and are annotated with the respective metabolite name, while gray circles indicate volatile metabolites whose presence was not significantly different.

**FIGURE 8:**
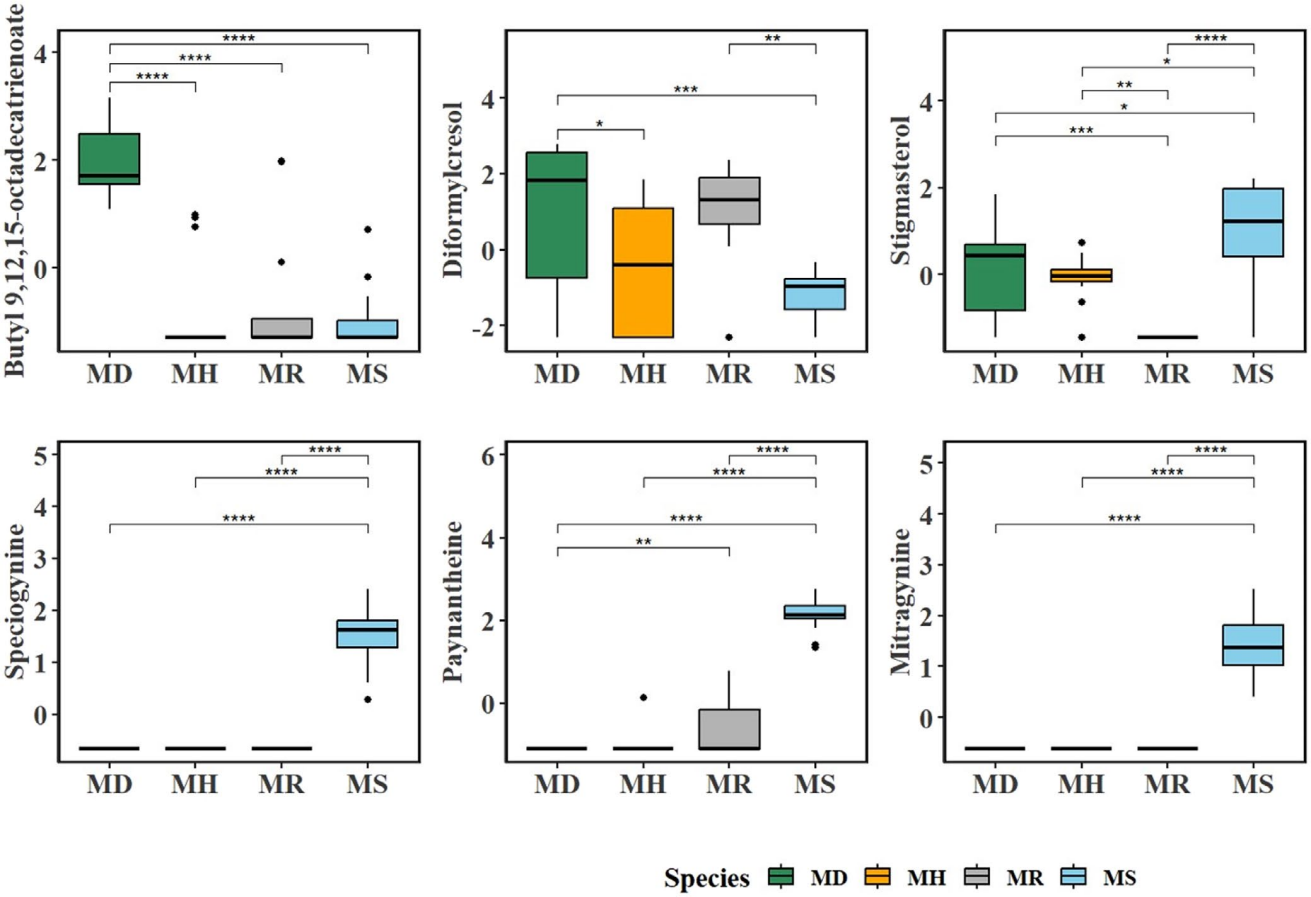
Boxplots of abundance of volatile metabolites that were significant in differentiating between the four *Mitragyna* species (*Mitragyna diversifolia* (Wall. ex G.Don) Havil. or MD, *Mitragyna hirsuta* Havil. or MH, *Mitragyna rotundifolia* (Roxb.) Kuntze or MR, and *Mitragyna speciosa* (Korth.) Havil. or MS) as well as significantly loaded the principal axes in the PCA plot. The bar plots include normalized values (mean ± one SD), with the boxes ranging from the 25% and the 75% percentiles, 5% and 95% percentiles indicated as error bars, and horizontal lines within boxes indicating median values, while the dots indicate outliers. The asterisks in various subplots indicate statistically significant difference in qualitative presence of the given metabolite between the four species.

Figure 7 includes volcano plots containing volatile metabolites with significant differential presence across each *Mitragyna* species compared to the three remaining species, with Figure 8 containing the boxplots exhibiting the abundance of these volatile metabolites. Volatile metabolites that were significant discriminators (as per PLS‐DA) and exhibited a significant fold change are highlighted in red (indicating higher presence in a given species) or green (indicating lower presence in a given species) circles. The log_2_ fold change cutoff values were set at 2 and −2 (fold change > 4) and 2 for the −log FDR adjusted *p*‐value (< 0.01) to identify volatile metabolites with the largest differences. Notably, no volatile metabolites with a significantly differential presence were identified in *M. hirsuta* , while the presence of paynantheine was lower in both *M. rotundifolia* and *M. diversifolia* . We also observed a higher presence of butyl 9,12,15‐octadecatrienoate in *M. diversifolia* , as indicated in Figure 8. Despite kratom having the lowest overall number of secondary volatile metabolites among the four species, its profile had the highest number of discriminating volatile metabolites with differential presence. Five volatile metabolites had a higher presence in kratom (paynantheine, speciogynine, mitragynine, speciociliatin, and stigmasterol), while the presence of two volatile metabolites (corresponding to diformylcresol and butyl 9,12,15‐Octadectrienoate) was lower.

## 4 Discussion

Ecological and evolutionary factors play an important role in shaping the production, diversity, and interspecific regulation of secondary metabolites (Xu and Gaquerel 2025). The present study used untargeted metabolomics to qualitatively analyze the presence or absence of secondary volatile metabolites as a function of environmental variables in four *Mitragyna* species growing naturally in various regions of Thailand. Volatile metabolites that significantly loaded the first two principal axes of the unsupervised PCA were first identified, while key volatile metabolites important in discriminating between the four species were identified through a supervised PLS‐DA. Further analyses using boxplots and volcano plots were conducted to explore and compare the presence of these volatile metabolites, aiming to elucidate any interspecific differences.

Photosynthesis leads to the production of primary metabolites involved with the basic cellular functions related to growth, development, or reproduction of a plant. Secondary metabolites are derived from primary metabolites through various biochemical pathways as a response to various environmental pressures, such as herbivory, pathogen attack, UV radiation, and competition with other plants (Leksungnoen et al. 2025; Qaderi et al. 2023). Environmental factors can influence the production of primary metabolites, and hence directly affect the presence of SMs (Gago et al. 2016). For example, high light intensity has been reported to increase photosynthesis and subsequently mitragynine concentration in kratom (Leksungnoen et al. 2022). In the present study, kratom demonstrated traits indicative of higher photosynthetic efficiency, such as high SLA, CHL, and ST.

The mentioned traits suggest a higher growth rate and potential for greater production of primary metabolites, which could translate into increased synthesis of secondary metabolites. Conversely, *M. diversifolia* , with the smallest LA, lowest SLA, CHL, and ST, would have a relatively slower growth rate and potentially lower production of bioactive compounds. Such variations in leaf traits and photosynthetic capacity underscore the influence of genetic and environmental factors on metabolite profiles within the *Mitragyna* genus. Various leaf functional traits such as LA, SLA, and CHL are closely linked with growth and defense strategies (Kergunteuil et al. 2018; Vleminckx et al. 2018), as have *F*_v_/*F*_m_ and PI (Leksungnoen et al. 2022).

SLA, a structural defense trait influencing leaf palatability and metabolic activity, decreases under abiotic stress conditions to enhance leaf strength and resistance to herbivory (de Sena et al. 2021; Poorter et al. 2004). Our study corroborates these findings, exhibiting a negative correlation between SLA and the number of secondary volatile metabolites. On the contrary, the association of a higher CHL content with a higher presence of secondary volatile metabolites diverged from the understanding of CHL's role in phenolic compound abundance (Selvaraj and Sankar 2010). A significant positive correlation between leaf pH and the presence of secondary volatile metabolites is indicative of the influence of leaf pH on the adaptive defense mechanisms against stresses, previously linked to defense mechanisms against drought, heat, and salinity stresses (Cornelissen et al. 2006; Meyer et al. 2021). In other words, a less acidic leaf pH, indicative of stress conditions, is associated with higher secondary metabolite abundance (Meyer et al. 2021).

Previous studies have demonstrated that environmental conditions and genetic differences profoundly influence the secondary metabolite profiles across plant species within the Rubiaceae family (Rastogi et al. 2020). For instance, differential accumulation of phenolic compounds in species of the *Ocimum* genus has been linked to their role in defense against environmental stresses (Rastogi et al. 2020). Similarly, variations in pyrrolizidine alkaloid levels between *Senecio jacobaea* and *S. aquaticus* underscore the environmental influence on secondary metabolite production (Van Dam and Hare 1998). Moreover, unique metabolite profiles contribute to species‐specific immune responses, where metabolic regulation plays a crucial role in shaping plant defense strategies (Piasecka et al. 2015).

Stresses such as seasonal drought can significantly impact the presence of specific secondary metabolites involved in oxidative stress protection, such as phenolics and flavonoids (Klem et al. 2015; Zas and Fernández‐López 2005). Variations in VPD have been shown to influence the foliar metabolite profile, affecting both primary and secondary metabolites, thereby modulating plant resilience to environmental stresses (Lihavainen et al. 2016). Additionally, drought intensity has been reported to increase the production of secondary metabolites in red betel leaves, reflecting adaptive responses to varying water availability (Lailaty et al. 2021). In our analysis, we observed an increased average presence of SMs with higher drought fluctuations, as indicated by the Palmer Drought Severity Index (PDSI) and mean VPD (Figures 3 and 4).

Various species in the Rubiaceae family have been reported to contain secondary metabolites such as triterpenoid saponins, C17‐methylated corynanthe monoterpene indole alkaloids, and their derivatives. These include compounds such as monoterpenoid indole alkaloids (Ahmad et al. 2022; Bakrim et al. 2022) such as including ajmalicine, phytosterols such as stigmasterol, and stigmastanes such as gamma sitosterol (Ahmad et al. 2022). Apart from these alkaloids, other secondary metabolites such as flavonoids, saponins, monoterpenes, triterpenoids, secoiridoids, and polyphenolic compounds have also been detected in *Mitragyna* species (Raffa 2014). Reviews of leaf secondary metabolites in 10 *Mitragyna* species indicated that alkaloids are the most prevalent secondary metabolites, and while most species contain 5–10 alkaloids, kratom and *M. parvifolia* had a relatively higher number of alkaloids (34 and 18, respectively) (Ahmad et al. 2022; Brown et al. 2017).

In our study, kratom had the lowest presence of secondary volatile metabolites relative to the other three allied species, potentially contributing to a higher growth rate as indicated by its leaf traits (Table 1). Interspecific variation of secondary metabolites in the *Mitragyna* species such as kratom and *M. diversifolia* exhibits distinct alkaloid profiles, largely influenced by their genetic makeup (León et al. 2009; Martins and Nunez 2015). For instance, kratom is characterized by a higher presence of mitragynine, distinguishing it from other species (Kamble et al. 2020). Additionally, indole alkaloids such as ajmalicine are differentially distributed among the *Mitragyna* species, further emphasizing the interspecific chemical diversity within this genus (León et al. 2009). In the present study, ajmalicine was mostly detected in *M. diversifolia* and one tree of *M. rotundifolia* but was absent in both *M. hirsuta* and MS.

Corresponding with a recent study by Sudmoon et al. (2025), it was observed that coumaran, palmitic acid, and phytol were consistently present in all the trees of the four species in our study. Sudmoon et al. (2025) focused on quantitative analysis of the four species in the NE part of Thailand, while the present study was a qualitative analysis of secondary volatile metabolites in *Mitragyna* species across multiple regions of Thailand to capture environmental influence on foliar metabolome. Further correspondence was observed for squalene, phytol, and vitamin E, identified in all the species in both studies, as well as 2,4‐Di‐tert‐butylphenol (identified in both studies in *M. hirsuta* and *M. rotundifolia* ). Broader interspecific variations were observed in mitragynine (found in *M. diversifolia* and kratom by Sudmoon et al. (2025) but only in kratom in the present study), paynantheine (only in kratom by Sudmoon et al. (2025) but in *M. hirsuta* , *M. rotundifolia* , and kratom in the present study), and dodecanoic acid (identified in all species by previous authors but only in *M. hirsuta* , *M. rotundifolia* , and *M. diversifolia* in our study), whereas octadecane was present in *M. rotundifolia* , *M. hirsuta* , and kratom in Sudmoon et al. (2025) but only in *M. diversifolia* in the current study. Mitraphylline (identified in all four species) was identified in the foliar metabolome of *M. diversifolia* , *M. rotundifolia* , and *M. hirsuta* in our analysis, while the epimer of rhynchophylline (isorhynchophylline, detected in only *M. rotundifolia* ) was present in *M. diversifolia* , *M. rotundifolia* , and *M. hirsuta* in our analysis.

From within a pool of 409 volatile metabolites, we identified 10 important interspecific discriminators for the four species, with only seven volatile metabolites significantly loading the first two PCA axes and are briefly discussed. The results of PLS‐DA highlighted specific SMs that effectively discriminated between the four *Mitragyna* species (Figure 4). While research on metabolites present in *Mitragyna* species dates back to 1939 (Barger et al. 1939) and various techniques have been employed for metabolite identification and quantification (Laforest et al. 2023; Veeramohan et al. 2023), most emphasis has been on kratom. The presence of mitragynine, speciogynine, stigmasterol, and paynantheine was significantly higher in kratom, with mitragynine being the most important in distinguishing kratom from the other species, while butyl 9,12,15‐octadecatrienoate was found to be significantly higher in *M. diversifolia* . Butyl 9,12,15‐octadecatrienoate was more frequently found in *M. diversifolia* and was a significant discriminator between *M. diversifolia* and the remaining species. This chemical is an esterified form of linolenic acid and plays a role in lipid metabolism and biosynthesis of signaling molecules related to plant defense and development and has been reported to have anti‐inflammatory properties (Devakumar et al. 2017).

Diformylcresol, a phenolic compound, was detected in all species but was a good discriminator of *M. rotundifolia* , *M. hirsuta* , and *M. diversifolia* from kratom. The presence of similar phenolic aldehydes and compounds has often been attributed to biotic and abiotic stresses (Kumar et al. 2023). This compound is also utilized as a precursor in the synthesis of various compartmental ligands (Sönmez et al. 2010). Stigmasterol, a phytosterol commonly found in the Rubiaceae family, has been identified in various *Mitragyna* species such as *M. rotundifolia* (Kang et al. 2006) and kratom (Phongprueksapattana et al. 2008). Known for its neuroprotective, anticancer, anti‐inflammatory, anti‐diabetic, immunomodulatory, and antioxidant properties (Bakrim et al. 2022), stigmasterol was notably absent in *M. rotundifolia* samples but its presence was higher in kratom. This difference indicated stigmasterol as a significant discriminator between these three species and *M. rotundifolia* .

The presence of paynantheine, speciogynine, and mitragynine was notable in kratom, as depicted in the boxplot (Figure 6). Paynantheine, an oxindole alkaloid, and speciociliatine, a diastereomer of mitragynine, were identified among the most prevalent metabolites in kratom (Takayama 2004). Monoterpene indole alkaloids, including derivatives of paynantheine, have been previously documented in various parts of *M. diversifolia* (Cao et al. 2013), known for their analgesic and anti‐inflammatory properties (Boffa et al. 2018). Additionally, both paynantheine and speciogynine have been noted for their role as low‐potency, competitive antagonists at opioid receptors (León et al. 2009). Isopaynantheine, structurally similar to other significant alkaloids found in kratom and a diastereomer of paynantheine (Philipp et al. 2011), has also been identified for its potential as an opioid receptor agonist (Chakraborty et al. 2021).

In the current study, the presence of multiple isomeric alkaloids was identified in *Mitragyna* species. The differentiation of mitragynine, speciogynine, and speciociliatine in this study is therefore considered tentative, given the limited capacity of GC–MS to resolve these structural isomers, particularly mitragynine and speciociliatine, which require confirmation using orthogonal techniques, such as Quadrupole Time‐of‐Flight Mass Spectrometry/Mass Spectrometry (QTOF‐MS/MS) and NMR, and authentic standards (Wang et al. 2014). Similarly, the apparent detection of isopaynantheine in *M. hirsuta* and *M. rotundifolia* warrants further investigation to determine whether it reflects species‐specific biosynthetic variation, post‐harvest metabolite transformation, or analytical limitations associated with the untargeted GC–MS workflow. While these identifications are putative, the comparative metabolite patterns observed across species remain valid and provide a basis for further targeted biochemical and taxonomic research.

## 5 Conclusions

Although *Mitragyna* species are widely reported for their pharmacologically active compounds, information pertaining to the interspecific differences in the metabolomic diversity of four *Mitragyna* species ( *M. diversifolia* , *M. hirsuta* , *M. rotundifolia* , and *M. speciosa* ) is largely unavailable. In this study, we reported the interspecific presence of secondary volatile metabolites unique to these four naturally occurring species in Thailand and examined the influence of environmental factors on the presence of these secondary metabolites. The presence of secondary volatile metabolites was influenced by a combination of warmer temperatures, increased air vapor pressure deficit, reduced rainfall (drought), and low wind speed. While metabolites such as γ‐Sitosterol, Vitamin E, Squalene, and Phytol were consistently present across the four species, significant differences were observed in key alkaloids like mitragynine, paynantheine, and dodecanoic acid. Correlation between photosynthetic efficiency (SLA, CHL) and metabolite accumulation was a potential indicator of metabolic trade‐offs between growth and defense strategies. Despite metabolic differences, PLS‐DA was unable to clearly separate the foliar metabolomes of *M. hirsuta* and *M. diversifolia* , suggesting profiles are either partially shared or that current resolution and sample variation limit discrimination at this level. Future research will focus on quantifying metabolite levels across regions to validate environmental effects, while employing advanced spectroscopy techniques to detect volatile and heavier bioactive compounds that may have not been identified in the current analysis.

## Funding

This work was financially supported by the Office of the Ministry of Higher Education, Science, Research and Innovation; and the Thailand Science Research and Innovation through the Kasetsart University Reinventing University Program, 2022. Funding for this work was kindly given by the Science, Research and Innovation Promotion Fund of the Thailand Science Research and Innovation (TSRI) through the Kasetsart University Research and Development Institute (KURDI). Project No. FF(KU) 9.64.

## Ethics Statement

The authors have nothing to report.

## Consent

The authors have nothing to report.

## Conflicts of Interest

The authors declare no conflicts of interest.

## Supporting information


**Data S1:** pei370118‐sup‐0001‐DataS1.docx.

**Data S2:** pei370118‐sup‐0002‐DataS2.zip.

## Data Availability

The data that support the findings of this study are openly available in *figshare* located at 10.6084/m9.figshare.31025902.
