# Supplementary material for: Environmental Influence on the Untargeted Foliar Metabolome of Naturally Growing Mitragyna Species in Thailand
Source: Plant Environ Interact. 2026 Jan 28;7(1):e70118. doi: 10.1002/pei3.70118 (PMC12848527; doi:10.1002/pei3.70118)
Supplement: Supplementary file 1 — Data S1: pei370118‐sup‐0001‐DataS1.docx. [file PEI3-7-e70118-s002.docx]

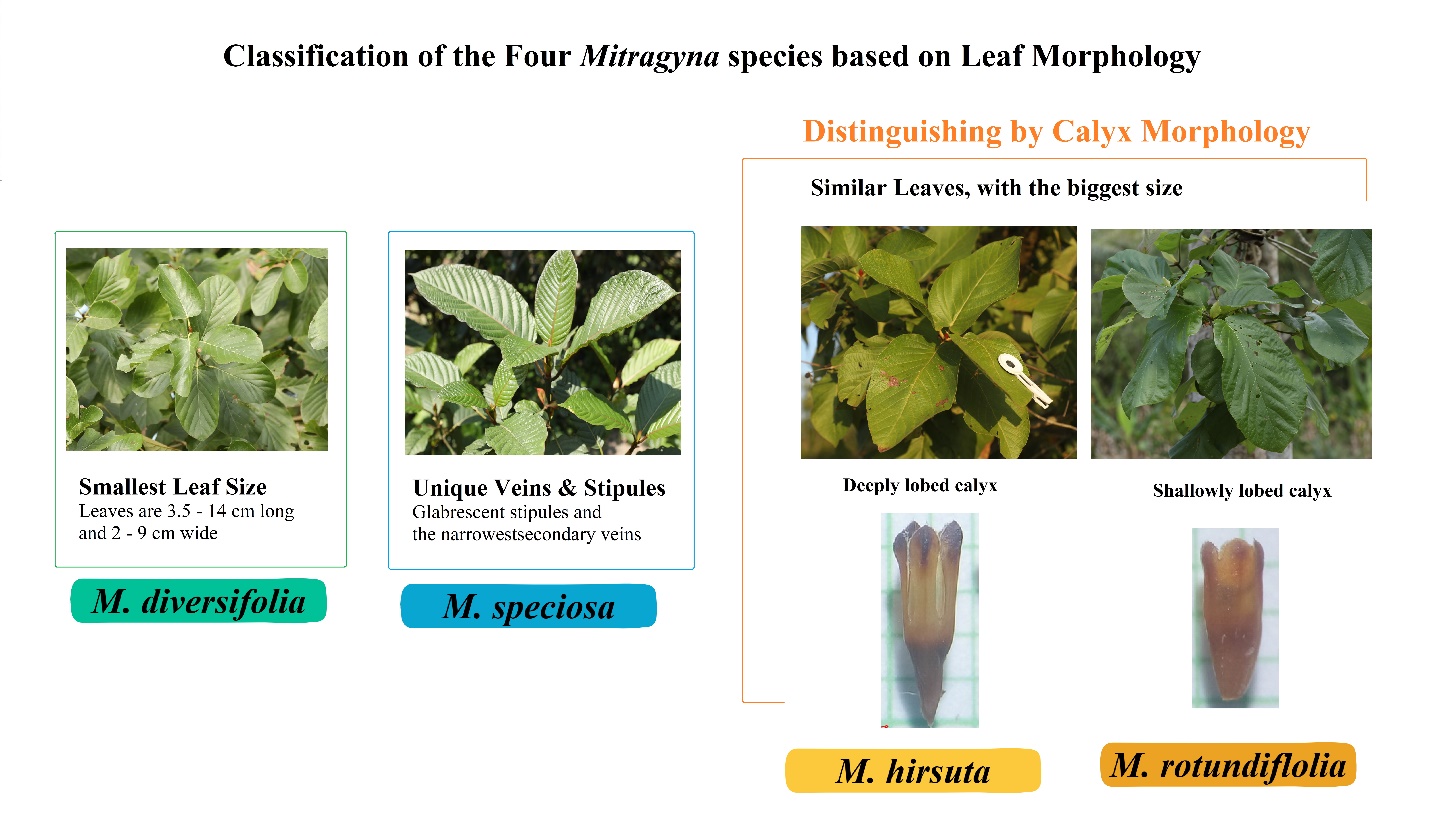


**Figure S1.** Representative leaf morphology used to differentiate the four *Mitragyna* species in Thailand. *M. diversifolia* is characterized by the smallest leaves, while *M. speciosa* is distinguished by glabrescent stipules and the narrowest secondary veins among the larger-leafed species. *M. hirsuta* and *M. rotundifolia* show highly similar leaf morphology and are most reliably separated by calyx morphology, with *M. hirsuta* exhibiting a deeply lobed calyx and *M. rotundifolia* a shallowly lobed calyx.

As seen in Figure S1, *M. diversifolia* can be distinguished by its comparatively small leaves, measuring 5–14 cm long and 2–9 cm wide. *M. speciosa* can be differentiated by glabrescent stipules and comparatively narrow secondary veins among the larger-leafed species. *M. hirsuta* and *M. rotundifolia* have similar leaf morphology and are most reliably separated by calyx morphology, with *M. hirsuta* showing a deeply lobed calyx and *M. rotundifolia* a shallowly lobed calyx. The interested readers are referred to Ngernsaengsaruay et al. (2022) for further details about the differences in morphology.

**Table S1.** Locations of Mitragyna species with tree characteristics. The four species are MD or *Mitragyna diversifolia* (Wall. ex G.Don) Havil*.,* MH or *M. hirsuta* Havil*.,* MR or *M. rotundifolia* (Roxb.) Kuntze*,* and MS or *M. speciosa* (Korth.) Havil. (kratom).

| **No.** | **Date of collection** | **Part of Thailand** | **Location** | | **Elevation (m)** | **Code name** | **Tree characteristics** | | | **Voucher specimen** |
| --- | --- | --- | --- | --- | --- | --- | --- | --- | --- | --- |
|  |  |  | **X** | **Y** |  |  | **Circumference at 1.30 m (cm)** | **Height (m)** | **Crown area (m^2^)** |  |
| ***Mitragyna diversifolia* (MD)** | | | | | | | | | | |
| 1 | 4 Nov. 2020 | Central (C) | 675117 | 1543502 | 17.0 | MD1 | 117.9 | 13.0 | 48.4 | *Ngernsaengsaruay et al. Md01-04112020* |
| 2 | 4 Nov. 2020 | Central (C) | 667512 | 1531680 | 26.0 | MD2 | 67.3 | 5.0 | 10.7 | *Ngernsaengsaruay et al. Md02-04112020* |
| 3 | 3 Dec. 2020 | Central (C) | 669771 | 1604821 | 8.0 | MD3 | 95.0 | 8.0 | 41.2 | *Ngernsaengsaruay et al. Md03-03122020* |
| 4 | 3 Dec. 2020 | Central (C) | 669787 | 1604809 | 6.0 | MD4 | 45.0 | 5.0 | 12.6 | *Ngernsaengsaruay et al. Md04-03122020* |
| 5 | 3 Dec. 2020 | Central (C) | 669791 | 1604762 | 4.0 | MD5 | 55.5 | 8.0 | 20.2 | *Ngernsaengsaruay et al. Md05-03122020* |
| 6 | 5 Dec. 2020 | East (E) | 791444 | 1547963 | 8.0 | MD6 | 83.4 | 12.5 | 42.4 | *Ngernsaengsaruay et al. Md06-05122020* |
| 7 | 5 Dec. 2020 | East (E) | 790816 | 1547655 | 7.0 | MD7 | 53.0 | 7.0 | 18.0 | *Ngernsaengsaruay et al. Md07- 05122020* |
| 8 | 5 Dec. 2020 | East (E) | 790817 | 1547644 | 7.0 | MD8 | 52.0 | 7.0 | 19.2 | *Ngernsaengsaruay et al. Md08- 05122020* |
| 9 | 5 Dec. 2020 | East (E) | 790844 | 1547640 | 7.5 | MD9 | 80.7 | 7.5 | 25.5 | *Ngernsaengsaruay et al. Md09- 05122020* |
| 10 | 5 Dec. 2020 | East (E) | 790873 | 1547661 | 13.0 | MD10 | 63.4 | 7.0 | 14.2 | *Ngernsaengsaruay et al. Md10- 05122020* |
| 11 | 19 Feb. 2021 | North (N) | 576346 | 1913972 | 67.0 | MD11 | 119.9 | 16 | 47.0 | *Ngernsaengsaruay et al. Md11-19022021* |
| 12 | 20 Feb. 2021 | North (N) | 533682 | 1841785 | 96.0 | MD13 | 93.8 | 12.0 | 40.2 | *Ngernsaengsaruay et al. Md13-20022021* |
| 13 | 21 Feb. 2021 | Central (C) | 533682 | 1802970 | 61.0 | MD14 | 110.0 | 8.0 | 30.3 | *Ngernsaengsaruay et al. Md14-21022021* |
| 14 | 21 Feb. 2021 | Central (C) | 583297 | 1771734 | 40.0 | MD15 | 24.0 | 8.0 | 1.4 | *Ngernsaengsaruay et al. Md15-21022021* |
| 15 | 26 Mar. 2021 | South (S) | 522874 | 1011406 | 4.0 | MD16 | 42.0 | 5.3 | 15.2 | *Ngernsaengsaruay et al. Md16-26032021* |
| 16 | 26 Mar. 2021 | South (S) | 522835 | 1011368 | 1.0 | MD17 | 93.6 | 5.4 | 36.8 | *Ngernsaengsaruay et al. Md17-26032021* |
| 17 | 26 Mar. 2021 | South (S) | 522834 | 1011370 | 3.0 | MD18 | 53.0 | 9.0 | 15.5 | *Ngernsaengsaruay et al. Md18-26032021* |
| 18 | 26 Mar. 2021 | South (S) | 522830 | 1011375 | 7.0 | MD19 | 143.0 | 10.0 | 87.4 | *Ngernsaengsaruay et al. Md19-26032021* |
| 19 | 26 Mar. 2021 | South (S) | 522854 | 1011392 | 6.0 | MD20 | 86 | 9.0 | 34.7 | *Ngernsaengsaruay et al. Md20-26032021* |
| ***Mitragyna rotundifolia* (MR)** | | | | | | | | | | |
| 20 | 4 Dec. 2020 | Northeast (NE) | 812909 | 1640338 | 235.0 | MR1 | 134.6 | 11.8 | 94.6 | *Ngernsaengsaruay et al. Mr01-04122020* |
| 21 | 21 Feb. 2021 | Central (C) | 583297 | 1771734 | 49.0 | MR2 | 67.0 | 12.0 | 23.8 | *Ngernsaengsaruay et al. Mr02-21022021* |
| 22 | 21 Feb. 2021 | North (N) | 649144 | 1701912 | 30.0 | MR3 | 52.0 | 8.0 | 19.6 | *Ngernsaengsaruay et al. Mr03-21022021* |
| 23 | 22 Feb. 2021 | Central (C) | 712495 | 1667975 | 70.0 | MR4 | 136.0 | 12.0 | 80.9 | *Ngernsaengsaruay et al. Mr04-22022021* |
| 24 | 22 Feb. 2021 | Central (C) | 712495 | 1667975 | 70.0 | MR5 | 55.0 | 12.0 | 64.7 | *Ngernsaengsaruay et al. Mr05-22022021* |
| 25 | 22 Feb. 2021 | Central (C) | 712495 | 1667975 | 70.0 | MR6 | 24.0 | 6.0 | 21.7 | *Ngernsaengsaruay et al. Mr06-22022021* |
| 26 | 22 Feb. 2021 | Central (C) | 700222 | 1678227 | 60.0 | MR7 | 126.0 | 8.0 | 42.2 | *Ngernsaengsaruay et al. Mr07-22022021* |
| 27 | 27 Mar. 2021 | South (S) | 523162 | 1187713 | 45.0 | MR8 | 96.8 | 12.0 | 37.3 | *Ngernsaengsaruay et al. Mr08-27032021* |
| 28 | 27 Mar. 2021 | South (S) | 523165 | 1187711 | 40.0 | MR9 | 129.8 | 14.0 | 64.7 | *Ngernsaengsaruay et al. Mr09-27032021* |
| 29 | 27 Mar. 2021 | South (S) | 523120 | 1187685 | 41.0 | MR10 | 92.0 | 19.0 | 40.4 | *Ngernsaengsaruay et al. Mr10-27032021* |
| 30 | 27 Mar. 2021 | South (S) | 523215 | 1187709 | 35.0 | MR11 | 130.0 | 20.0 | 67.2 | *Ngernsaengsaruay et al. Mr11-27032021* |
| 31 | 27 Mar. 2021 | South (S) | 523256 | 1187686 | 49.0 | MR12 | 15.0 | 5.0 | 3.6 | *Ngernsaengsaruay et al. Mr12-27032021* |
| ***Mitragyna hirsuta* (MH)** | | | | | | | | | | |
| 32 | 3 Dec. 2020 | Northeast (NE) | 816673 | 1606055 | 345.0 | MH1 | 78.4 | 12.5 | 28.3 | *Ngernsaengsaruay et al. Mh01- 04122020* |
| 33 | 3 Dec. 2020 | Northeast (NE) | 816703 | 1606043 | 344.0 | MH2 | 62.0 | 11.0 | 22.3 | *Ngernsaengsaruay et al. Mh02- 04122020* |
| 34 | 3 Dec. 2020 | Northeast (NE) | 816708 | 1606066 | 342.0 | MH3 | 63.6 | 12.5 | 15.9 | *Ngernsaengsaruay et al. Mh03- 04122020* |
| 35 | 3 Dec. 2020 | Northeast (NE) | 812793 | 1667815 | 188.0 | MH4 | 50.0 | 5.7 | 20.1 | *Ngernsaengsaruay et al. Mh04-04122020* |
| 36 | 19 Feb. 2021 | North (N) | 534929 | 1943769 | 260.0 | MH5 | 71.0 | 11.0 | 42.9 | *Ngernsaengsaruay et al. Mh05-19022021* |
| 37 | 19 Feb. 2021 | North (N) | 576066 | 1913988 | 71.0 | MH6 | 90.0 | 14.0 | 56.5 | *Ngernsaengsaruay et al. Mh06-19022021* |
| 38 | 20 Feb. 2021 | North (N) | 507925 | 1890589 | 143.0 | MH7 | 155.0 | 12.0 | 57.5 | *Ngernsaengsaruay et al. Mh07-20022021* |
| 39 | 22 Feb. 2021 | Central (C) | 701418 | 1531446 | 10.0 | MH8 | 122.0 | 22.0 | 42.0 | *Ngernsaengsaruay et al. Mh08-22022021* |
| 40 | 20 Feb. 2021 | North (N) | 576513 | 1909502 | 62.0 | MH9 | 70.0 | 9.1 | 24.2 | *Ngernsaengsaruay et al. Mh09-20022021* |
| 41 | 10 July 2021 | Central (C) | 679652 | 1514031 | 7.0 | MH10 | 99.5 | 9.0 | 72.4 | *Ngernsaengsaruay et al. Mh10-09072021* |
| 42 | 10 July 2021 | Central (C) | 679635 | 1514035 | 7.0 | MH11 | 58 | 10.0 | 15.5 | *Ngernsaengsaruay et al. Mh11-09072021* |
| 43 | 10 July 2021 | Central (C) | 679631 | 1514026 | 7.0 | MH12 | 69.5 | 9.0 | 9.62 | *Ngernsaengsaruay et al. Mh12-09072021* |
| 44 | 10 July 2021 | Central (C) | 679631 | 1514018 | 7.0 | MH13 | 53.5 | 9.0 | 26.0 | *Ngernsaengsaruay et al. Mh13-09072021* |
| ***Mitragyna speciosa* (MS)** | | | | | | | | | | |
| 45 | 26 July 2021 | Central (C) | 670341 | 1565337 | 12.0 | MS1 | 57.0 | 7.7 | 28.7 | *Ngernsaengsaruay et al. Ms01-26092021* |
| 46 | 26 July 2021 | Central (C) | 670372 | 1565341 | 5.0 | MS2 | 22.0 | 4.0 | 13.9 | *Ngernsaengsaruay et al. Ms02-26092021* |
| 47 | 26 July 2021 | Central (C) | 670314 | 1565344 | 2.0 | MS3 | 51.0 | 7.7 | 15.6 | *Ngernsaengsaruay et al. Ms03-26092021* |
| 48 | 26 July 2021 | Central (C) | 670106 | 1565114 | 11.0 | MS4 | 54.0 | 4.0 | 11.8 | *Ngernsaengsaruay et al. Ms04-26092021* |
| 49 | 26 July 2021 | Central (C) | 670350 | 1565320 | 10.0 | MS5 | 74.2 | 8.0 | 21.9 | *Ngernsaengsaruay et al. Ms05-26092021* |
| 50 | 21 Oct. 2021 | South (S) | 440938 | 1041507 | 0 | MS6 | 61.0 | 10.7 | 30.7 | *Ngernsaengsaruay et al. Ms06-21102021* |
| 51 | 21 Oct. 2021 | South (S) | 439794 | 1039142 | 0 | MS7 | 92.0 | 14.0 | 41.8 | *Ngernsaengsaruay et al. Ms07-21102021* |
| 52 | 21 Oct. 2021 | South (S) | 440806 | 1036798 | 31.0 | MS8 | 85.0 | 14.5 | 12.9 | *Ngernsaengsaruay et al. Ms08-21102021* |
| 53 | 21 Oct. 2021 | South (S) | 441830 | 1036749 | 39.0 | MS9 | 31.5 | 9.7 | 15.9 | *Ngernsaengsaruay et al. Ms09-21102021* |
| 54 | 21 Oct. 2021 | South (S) | 439640 | 1038369 | 30.0 | MS10 | 82.0 | 15.0 | 40.7 | *Ngernsaengsaruay et al. Ms10-21102021* |
| 55 | 24 Nov. 2021 | Northeast (NE) | 405291 | 1859013 | 317.0 | MS11 | 25.3 | 5.0 | 15.9 | *Ngernsaengsaruay et al. Ms11-24112021* |
| 56 | 24 Nov. 2021 | Northeast (NE) | 405287 | 1859014 | 321.0 | MS12 | 16.0 | 5.1 | 2.7 | *Ngernsaengsaruay et al. Ms12-24112021* |
| 57 | 24 Nov. 2021 | Northeast (NE) | 405295 | 1859007 | 319.0 | MS13 | 15.8 | 4.0 | 7.8 | *Ngernsaengsaruay et al. Ms13-24112021* |
| 58 | 24 Nov. 2021 | Northeast (NE) | 405269 | 1859029 | 309.0 | MS14 | 15.5 | 4.0 | 3.0 | *Ngernsaengsaruay et al. Ms14-24112021* |
| 59 | 24 Nov. 2021 | Northeast (NE) | 405278 | 1859024 | 304.0 | MS15 | 18.0 | 5.0 | 5.5 | *Ngernsaengsaruay et al. Ms15-24112021* |
| 60 | 18 Dec. 2021 | North (N) | 496848 | 2057407 | 292.0 | MS16 | 22.0 | 6.0 | 8.1 | *Ngernsaengsaruay et al. Ms16-18122021* |
| 61 | 18 Dec. 2021 | North (N) | 498869 | 2057325 | 303.0 | MS17 | 35.0 | 7.0 | 12.5 | *Ngernsaengsaruay et al. Ms17-18122021* |
| 62 | 18 Dec. 2021 | North (N) | 498826 | 2059233 | 290.0 | MS18 | 40.0 | 3.3 | 15.4 | *Ngernsaengsaruay et al. Ms18-18122021* |
| 63 | 18 Dec. 2021 | North (N) | 493746 | 2113200 | 361.0 | MS19 | 14.5 | 5.2 | 3.8 | *Ngernsaengsaruay et al. Ms19-18122021* |

**
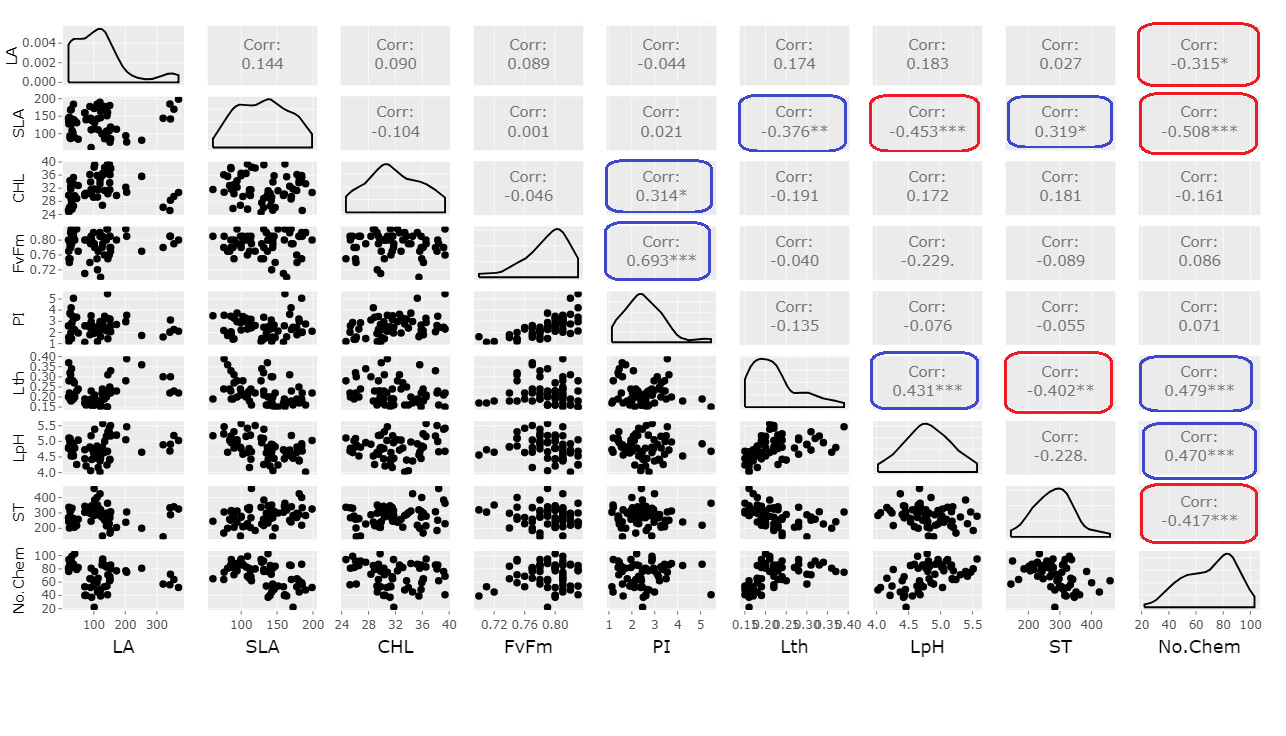
**

**Figure S2.** Pairwise Pearson correlation matrix among the measured leaf functional traits, photosynthetic parameters, and phytochemical traits of kratom seedlings. Scatterplots show bivariate relationships, with density plots shown along the diagonal. Significant correlation coefficients are annotated with asterisks (*=*P-value*  < 0.05, ***=P-value*  < 0.01, ****=P-value*  < 0.001). Blue boxes highlight significant positive correlations, and red boxes indicate significant negative correlations. Trait abbreviations: LA = Leaf Area, SLA = Specific Leaf Area, CHL = Chlorophyll Content, Fv/Fm = Maximum Quantum Yield of PSII, PI = Performance Index, Lth = Leaf Thickness, LpH = Leaf pH, ST = Stomatal Density, No.Chem = Number of Detected Leaf Chemicals.

**Table S2**. Leaf functional traits of *Mitragyna* species in various parts of Thailand. The four species are MD or *Mitragyna diversifolia* (Wall. ex G.Don) Havil*.,* MH or *M. hirsuta* Havil*.,* MR or *M. rotundifolia* (Roxb.) Kuntze*,* and MS or *M. speciosa* (Korth.) Havil. (kratom).

| **No.** | **Code name** | **Part of Thailand** | **Leaf traits (Average ± standard deviation)** | | | | | | | |
| --- | --- | --- | --- | --- | --- | --- | --- | --- | --- | --- |
|  |  |  | **Leaf area (LA)**  **(m^2^)** | **Specific leaf area (SLA)**  **(g m^-2^)** | **Chlorophyll content (CHL)**  **(SPAD)** | **Quantum yield (Fv/Fm)**  **(unitless)** | **Performance index (PI)**  **(unitless)** | **Leaf thickness**  **(mm)** | **Leaf pH**  **(unitless)** | **Stomatal density (SD)**  **(No. mm^-2^)** |
| 1 | MD1 | Central (C) | 31.97±4.37 | 239.37±46.84 | 37.78±2.16 | 0.83±0.01 | 4.64±0.42 | 0.27±0.05 | 4.62±0.08 | 184.80±29.84 |
| 2 | MD2 | Central (C) | 29.47±4.81 | 128.64±11.03 | 27.58±2.42 | 0.79±0.01 | 1.45±0.32 | 0.34±0.05 | 4.96±0.05 | 228.67±10.29 |
| 3 | MD3 | Central (C) | 26.61±3.29 | 167.55±22.5 | 32.60±2.38 | 0.82±0 | 3.62±0.74 | 0.31±0.11 | 4.72±0.06 | 252.93±19.80 |
| 4 | MD4 | Central (C) | 28.23±5.75 | 169.49±20.06 | 28.71±1.74 | 0.83±0.01 | 4.22±0.83 | 0.18±0.02 | 4.53±0.07 | 241.07±11.70 |
| 5 | MD5 | Central (C) | 35.58±7.37 | 184.25±32.04 | 35.26±3.62 | 0.82±0.01 | 5.07±1.35 | 0.19±0.01 | 4.92±0.2 | 219.87±31.00 |
| 6 | MD6 | East (E) | 21.16±3.23 | 127.68±10.33 | 24.63±2.55 | 0.75±0.01 | 1.19±0.27 | 0.28±0.09 | 5.11±0.24 | 336.60±19.16 |
| 7 | MD7 | East (E) | 24.60±3.01 | 131.14±11.56 | 26.06±3.07 | 0.75±0.03 | 1.40±0.53 | 0.32±0.06 | 5.09±0.18 | 258.53±21.74 |
| 8 | MD8 | East (E) | 22.75±3.51 | 147.31±15.52 | 26.13±2.60 | 0.74±0.07 | 1.54±0.79 | 0.22±0.07 | 4.83±0.08 | 202.20±13.50 |
| 9 | MD9 | East (E) | 19.80±2.38 | 144.04±8.67 | 25.30±2.57 | 0.80±0.01 | 2.59±0.34 | 0.20±0.07 | 4.78±0.07 | 269.13±29.31 |
| 10 | MD10 | East (E) | 20.55±3.31 | 136.51±18 | 29.89±3.61 | 0.77±0.02 | 1.58±0.35 | 0.37±0.05 | 4.80±0.06 | 243.40±10.60 |
| 11 | MD11 | North (N) | 38.23±5.54 | 90.08±11.07 | 33.10±3.22 | 0.80±0.02 | 3.28±1.17 | 0.27±0.02 | 4.79±0.05 | 207.67±49.40 |
| 12 | MD13 | North (N) | 27.01±4.19 | 86.26±4.79 | 31.72±2.70 | 0.78±0.01 | 1.96±0.69 | 0.19±0.01 | 4.96±0.05 | 316.27±66.74 |
| 13 | MD14 | Central (C) | 24.48±3.42 | 94.28±14.39 | 34.30±4.15 | 0.79±0.01 | 3.73±1.08 | 0.20±0.02 | 4.98±0.20 | 312.53±37.39 |
| 14 | MD15 | Central (C) | 24.73±3.21 | 88.59±8.12 | 25.84±4.35 | 0.78±0.01 | 1.40±0.19 | 0.19±0.02 | 5.02±0.19 | 279.27±28.56 |
| 15 | MD16 | South (S) | 30.92±5.23 | 108.91±25.69 | 25.63±2.23 | 0.81±0.01 | 2.88±0.45 | 0.21±0.01 | 4.64±0.05 | 330.07±26.62 |
| 16 | MD17 | South (S) | 33.96±6.54 | 102.39±33.74 | 29.47±3.30 | 0.81±0.01 | 3.43±0.60 | 0.28±0.01 | 4.59±0.06 | 211.07±10.63 |
| 17 | MD18 | South (S) | 39.59±9.34 | 137.32±67.21 | 29.72±3.56 | 0.80±0.01 | 2.21±0.59 | 0.20±0.03 | 4.79±0.1 | 234.80±19.35 |
| 18 | MD19 | South (S) | 45.73±13.75 | 131.39±77.44 | 28.90±3.22 | 0.83±0.01 | 3.37±0.71 | 0.23±0.02 | 4.26±0.07 | 217.73±26.62 |
| 19 | MD20 | South (S) | 35.50±8.25 | 140.55±52.61 | 26.56±3.23 | 0.81±0.02 | 2.30±0.75 | 0.20±0.02 | 4.53±0.07 | 263.07±22.88 |
| 20 | MR1 | Northeast (NE) | 91.76±11.73 | 111.04±12.17 | 30.28±2.99 | 0.80±0.01 | 2.95±0.61 | 0.23±0.02 | 4.77±0.18 | 320.93±11.70 |
| 21 | MR2 | Central (C) | 134.47±30.01 | 85.57±11.37 | 38.30±3.69 | 0.80±0.02 | 3.45±0.96 | 0.23±0.02 | 5.00±0.06 | 252.20±20.07 |
| 22 | MR3 | North (N) | 128.45±26.32 | 99.95±8.12 | 36.57±3.50 | 0.79±0.02 | 2.89±1.15 | 0.21±0.04 | 5.57±0.12 | 278.00±42.31 |
| 23 | MR4 | Central (C) | 140.00±25.49 | 106.78±11.02 | 36.54±3.45 | 0.80±0.01 | 3.27±0.91 | 0.20±0.02 | 5.16±0.07 | 216.20±41.24 |
| 24 | MR5 | Central (C) | 127.49±14.11 | 98.98±18.98 | 26.80±2.99 | 0.81±0.02 | 2.87±0.92 | 0.24±0.01 | 4.81±0.07 | 150.00±19.83 |
| 25 | MR6 | Central (C) | 126.21±17.54 | 99.91±9.16 | 35.84±3.62 | 0.78±0.03 | 2.22±0.42 | 0.22±0.02 | 4.76±0.05 | 272.33±30.63 |
| 26 | MR7 | Central (C) | 151.56±17.61 | 75.75±9.1 | 36.12±4.68 | 0.78±0.02 | 2.17±0.55 | 0.24±0.01 | 5.24±0.04 | 169.13±22.64 |
| 27 | MR8 | South (S) | 343.35±77.47 | 141.86±55.4 | 28.35±2.05 | 0.81±0.01 | 3.11±0.39 | 0.30±0.03 | 4.91±0.10 | 288.20±45.30 |
| 28 | MR9 | South (S) | 320.00±109.99 | 143.86±75.7 | 26.17±1.77 | 0.78±0.01 | 1.59±0.31 | 0.30±0.04 | 4.89±0.03 | 144.87±12.30 |
| 29 | MR10 | South (S) | 354.19±109.12 | 169.94±95.95 | 29.47±3.53 | 0.79±0.01 | 2.26±0.36 | 0.23±0.02 | 5.19±0.08 | 340.40±29.07 |
| 30 | MR11 | South (S) | 341.57±104.08 | 185.00±62.76 | 25.24±1.73 | 0.81±0.01 | 2.03±0.42 | 0.22±0.02 | 4.69±0.07 | 333.00±35.420 |
| 31 | MR12 | South (S) | 368.91±138.8 | 198.72±103.38 | 30.73±2.09 | 0.80±0.01 | 2.11±0.27 | 0.22±0.02 | 5.03±0.10 | 324.80±67.89 |
| 32 | MH1 | Northeast (NE) | 102.83±45.17 | 131.55±57.3 | 39.18±3.69 | 0.78±0.02 | 2.46±0.54 | 0.21±0.03 | 5.39±0.33 | 384.4±42.41 |
| 33 | MH2 | Northeast (NE) | 153.24±53.95 | 119.47±26.13 | 38.19±3.02 | 0.77±0.03 | 2.63±0.86 | 0.22±0.04 | 5.51±0.03 | 186.47±19.57 |
| 34 | MH3 | Northeast (NE) | 149.14±49.32 | 108.65±15.78 | 39.39±2.72 | 0.76±0.03 | 2.30±0.78 | 0.22±0.02 | 5.42±0.16 | 229.47±27.09 |
| 35 | MH4 | Northeast (NE) | 144.74±27.91 | 89.96±18.83 | 30.35±2.39 | 0.75±0.03 | 1.43±0.45 | 0.31±0.02 | 5.31±0.41 | 273.08±63.89 |
| 36 | MH5 | North (N) | 252.06±70.1 | 81.10±14.77 | 35.62±4.02 | 0.77±0.02 | 1.73±0.50 | 0.36±0.07 | 4.65±0.05 | 200.53±36.74 |
| 37 | MH6 | North (N) | 153.60±24.83 | 85.82±15.61 | 37.96±3.32 | 0.78±0.02 | 2.07±0.69 | 0.33±0.05 | 5.17±0.07 | 308.20±29.62 |
| 38 | MH7 | North (N) | 204.43±43.41 | 76.24±5.46 | 30.80±2.76 | 0.81±0.01 | 3.55±0.86 | 0.39±0.03 | 5.47±0.10 | 306.07±25.24 |
| 39 | MH8 | Central (C) | 201.56±27.75 | 94.25±14.35 | 32.31±3.12 | 0.83±0.01 | 2.97±0.62 | 0.28±0.03 | 5.05±0.12 | 239.93±30.73 |
| 40 | MH9 | North (N) | 50.56±10.71 | 83.97±17.59 | 32.84±3.27 | 0.77±0.02 | 1.96±0.54 | 0.24±0.03 | 5.04±0.20 | 259.67±53.71 |
| 41 | MH10 | Central (C) | 150.65±20.90 | 180.40±40.00 | 33.66±2.00 | 0.81±0.01 | 3.21±0.54 | 0.25±0.01 | 4.40±0.27 | 290.33±34.42 |
| 42 | MH11 | Central (C) | 91.47±13.38 | 60.64±14.77 | 31.59±2.26 | 0.81±0.01 | 3.50±0.31 | 0.19±0.01 | 5.18±0.19 | 246.67±15.49 |
| 43 | MH12 | Central (C) | 151.09±22.47 | 113.58±26.60 | 31.54±2.45 | 0.81±0.01 | 3.17±0.54 | 0.24±0.02 | 4.43±0.14 | 268.13±18.93 |
| 44 | MH13 | Central (C) | 172.06±30.19 | 112.85±33.28 | 29.76±1.69 | 0.80±0.05 | 2.72±0.62 | 0.23±0.02 | 5.20±0.36 | 206.13±29.19 |
| 45 | MS1 | Central (C) | 71.69±16.33 | 160.14±35.408 | 30.23±2.94 | 0.80±0.02 | 2.61±0.70 | 0.19±0.06 | 4.44±0.17 | 343.20±44.08 |
| 46 | MS2 | Central (C) | 101.23±30.20 | 172.22±42.28 | 31.76±3.84 | 0.79±0.02 | 2.47±0.66 | 0.16±0.01 | 4.46±0.20 | 283.80±36.68 |
| 47 | MS3 | Central (C) | 99.23±28.55 | 135.03±23.79 | 29.25 2.59 | 0.82±0.00 | 1.91±0.69 | 0.16±0.01 | 4.63±0.22 | 298.40±22.91 |
| 48 | MS4 | Central (C) | 121.42±35.06 | 145.80±14.87 | 35.82±1.91 | 0.81±0.01 | 2.66±0.61 | 0.15±0.01 | 4.45±0.17 | 311.00±32.93 |
| 49 | MS5 | Central (C) | 141.89±64.71 | 151.28±28.27 | 31.25±4.86 | 0.83±0.01 | 2.13±0.58 | 0.15±0.02 | 4.24±0.14 | 300.20±45.88 |
| 50 | MS6 | South (S) | 119.68±14.90 | 189.71±28.51 | 33.38±3.48 | 0.82±0.01 | 2.75±0.75 | 0.16±0.01 | 4.02±0.06 | 283.80±22.05 |
| 51 | MS7 | South (S) | 93.35±20.79 | 137.52±12.04 | 31.20±3.10 | 0.80±0.02 | 2.50±0.76 | 0.16±0.01 | 4.42±0.15 | 345.80±29.23 |
| 52 | MS8 | South (S) | 119.87±10.64 | 180.85±36.33 | 34.56±4.37 | 0.81±0.02 | 2.99±1.35 | 0.17±0.02 | 4.37±0.14 | 424.80±14.13 |
| 53 | MS9 | South (S) | 143.66±9.19 | 160.91±20.03 | 39.38±5.33 | 0.83±0.01 | 5.46±2.09 | 0.15±0.01 | 4.68±0.10 | 362.40±37.61 |
| 54 | MS10 | South (S) | 75.47±4.36 | 140.16±15.23 | 30.45±3.24 | 0.80±0.01 | 2.60±0.86 | 0.16±0.02 | 4.21±0.06 | 315.40±48.52 |
| 55 | MS11 | Northeast (NE) | 87.62±12.68 | 177.72±12.50 | 36.17±1.78 | 0.74±0.03 | 1.80±0.33 | 0.18±0.01 | 4.82±0.07 | 297.20±38.00 |
| 56 | MS12 | Northeast (NE) | 111.82±17.36 | 142.45±7.37 | 29.30±1.04 | 0.72±0.02 | 1.18±0.15 | 0.18±0.02 | 4.65±0.03 | 351.06±21.85 |
| 57 | MS13 | Northeast (NE) | 110.29±7.16 | 184.24±31.92 | 35.92±1.29 | 0.75±0.03 | 2.00±0.34 | 0.18±0.01 | 4.85±0.28 | 400.20±22.80 |
| 58 | MS14 | Northeast (NE) | 121.69±8.03 | 163.31±23.60 | 35.50±1.08 | 0.70±0.02 | 1.62±0.21 | 0.17±0.01 | 4.56±0.07 | 319.80±43.08 |
| 59 | MS15 | Northeast (NE) | 71.45±5.28 | 158.89±16.49 | 29.80±1.06 | 0.71±0.03 | 1.13±0.14 | 0.17±0.02 | 4.73±0.05 | 305.20±31.69 |
| 60 | MS16 | North (N) | 133.81±18.57 | 177.33±32.03 | 31.33±2.63 | 0.76±0.02 | 2.45±0.36 | 0.19±0.02 | 4.64±0.11 | 271.00±30.52 |
| 61 | MS17 | North (N) | 113.90±7.86 | 111.53±14.80 | 31.52±2.44 | 0.77±0.02 | 2.40±0.63 | 0.18±0.02 | 4.16±0.22 | 334.4±40.08 |
| 62 | MS18 | North (N) | 89.97±14.67 | 144.21±25.54 | 33.93±2.09 | 0.79±0.02 | 2.39±0.35 | 0.16±0.02 | 4.06±0.14 | 300.4±49.33 |
| 63 | MS19 | North (N) | 101.17±8.83 | 114.30±11.08 | 38.44±2.50 | 0.79±0.01 | 2.42±0.27 | 0.16±0.01 | 4.67±0.11 | 458.6±44.77 |

**References**

Ngernsaengsaruay, Chatchai, Nisa Leksungnoen, Weereesa Boonthasak, Suwimon Utharatsamee, Phruet Racharak, Kantinan Leetanasakskul, Pornkanok Pongamorn, and Atcharaphan Saengbuapuean. "Additional knowledge on the genus Mitragyna (Rubiaceae) in Thailand." *Thai Forest Bulletin (Botany)* 50, no. 1 (2022): 20-39.
