# Supplementary material for: Environmental Influence on the Untargeted Foliar Metabolome of Naturally Growing Mitragyna Species in Thailand
Source: Plant Environ Interact. 2026 Jan 28;7(1):e70118. doi: 10.1002/pei3.70118 (PMC12848527; doi:10.1002/pei3.70118)
Supplement: Supplementary file 2 — Data S2: pei370118‐sup‐0002‐DataS2.zip. [file PEI3-7-e70118-s001.zip › pei370118-sup-0003-Supinfo3@Kratom_1.2_10mgml.pdf]

Qualitative result

Analyzed by	: Admin
Analyzed	: 26/9/2563 5:33:01
Sample Type	: Unknown
Level #	: 1
Sample Name	: Kratom_1.2
Sample ID	:
IS Amount	:
Sample Amount	: 1
Dilution Factor	: 1
Vial #	: 36
Injection Volume	: 1.00
Data File	: D:\NOC\Data+Batch\P'Farm\Kratom_1.2_01.qgd
Org Data File	: D:\NOC\Data+Batch\P'Farm\Kratom_1.2_01.qgd
Method File	: D:\NOC\Method\Kratom.qgm
Org Method File	: D:\NOC\Method\Kratom.qgm
Report File	:
Tuning File	: C:\GCMSsolution\System\Tune1\23-09-2563 N F1.qgt
Modified by	: Admin
Modified	: 5/10/2563 15:53:22

Chromatogram Kratom_1.2 D:\NOC\Data+Batch\P'Farm\Kratom_1.2_01.qgd

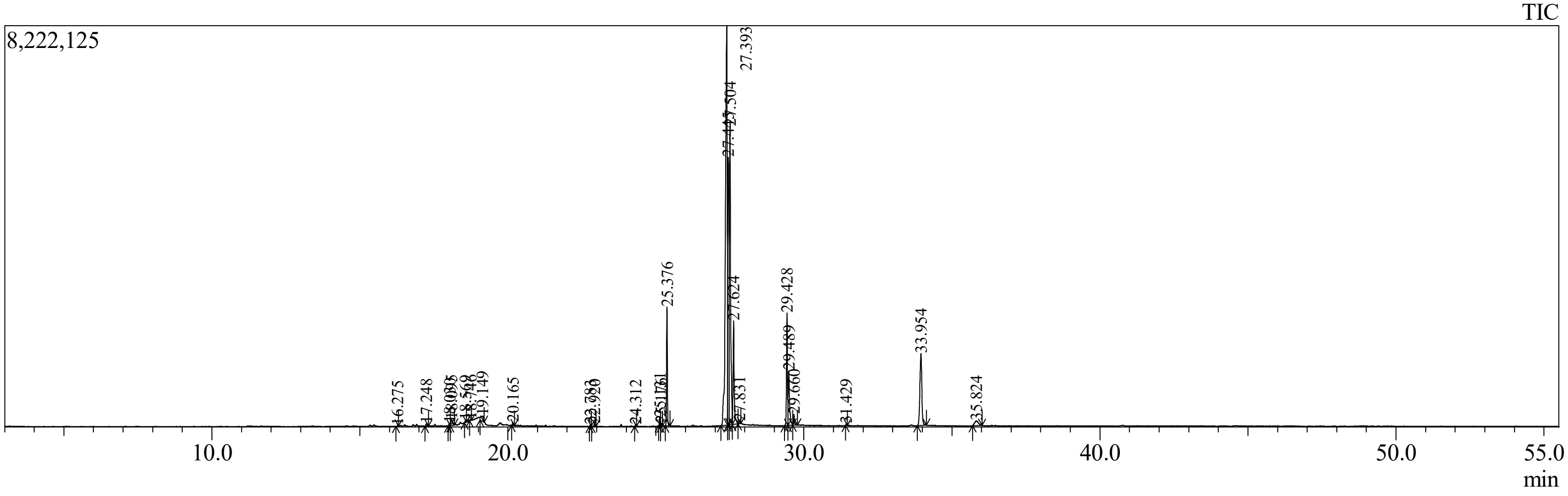

Library

<< Target >>
Line#:1 R.Time:16.275(Scan#:2656) MassPeaks:173
RawMode:Averaged 16.270-16.280(2655-2657) BasePeak:60.00(1754)
BG Mode:Calc. from Peak Group 1 - Event 1 Scan

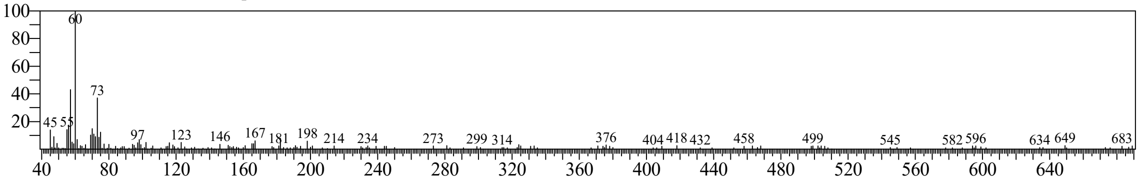

Hit#:1 Entry:36091 Library:NIST17.lib
SI:81 Formula:C6H10O5 CAS:498-07-7 MolWeight:162 RetIndex:1404
CompName:.beta.-D-Glucopyranose, 1,6-anhydro-

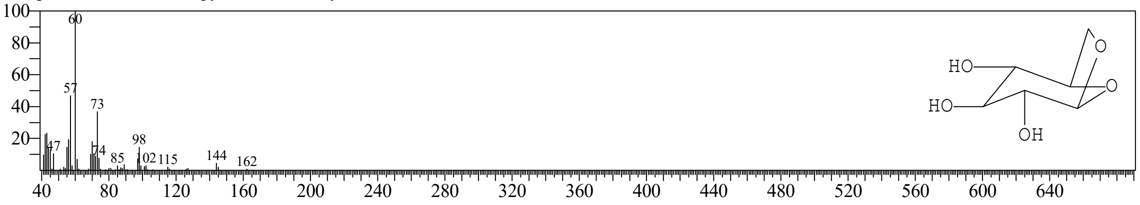

Hit#:2 Entry:51526 Library:NIST17.lib
SI:81 Formula:C6H12O6 CAS:2595-97-3 MolWeight:180 RetIndex:1698
CompName:D-Allose

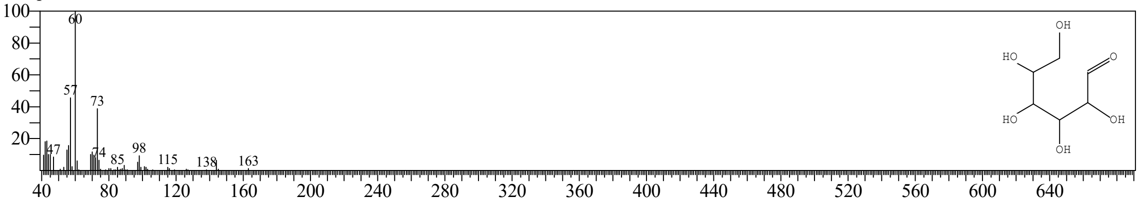

<< Target >>
Line#:2   R.Time:17.250(Scan#:2851) MassPeaks:174
RawMode:Averaged 17.245-17.255(2850-2852) BasePeak:73.05(5833)
BG Mode:Calc. from Peak Group 1 - Event 1 Scan

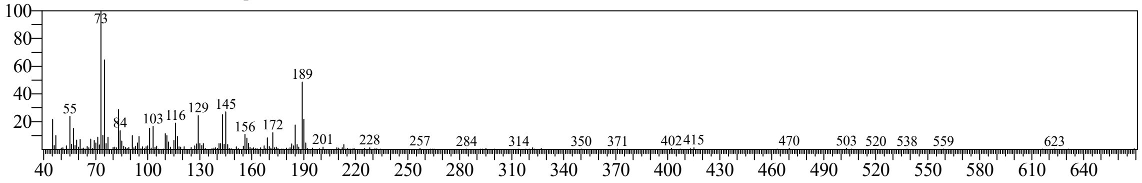

No hit compound

<< Target >>
Line#:3   R.Time:18.030(Scan#:3007) MassPeaks:135
RawMode:Averaged 18.025-18.035(3006-3008) BasePeak:73.05(1888)
BG Mode:Calc. from Peak Group 1 - Event 1 Scan

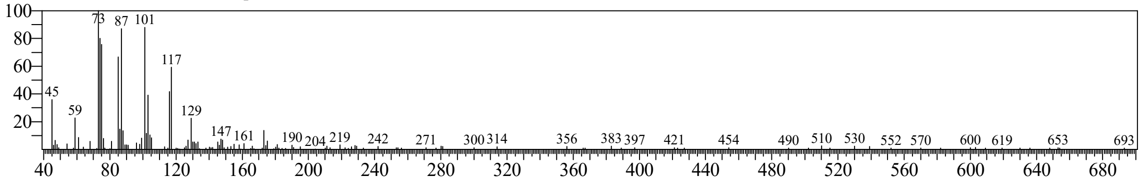

No hit compound

<< Target >>
Line#:4 R.Time:18.095(Scan#:3020) MassPeaks:150
RawMode:Averaged 18.090-18.100(3019-3021) BasePeak:60.00(12754)
BG Mode:Calc. from Peak Group 1 - Event 1 Scan

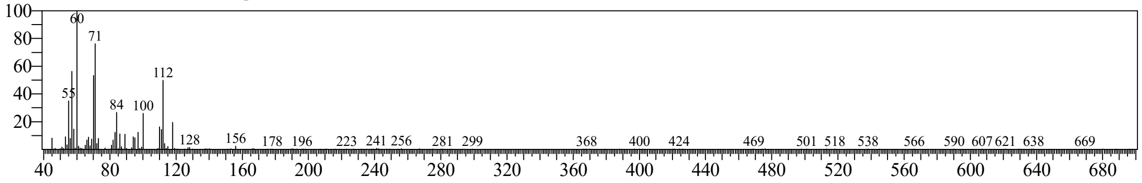

No hit compound

<< Target >>
Line#:5   R.Time:18.570(Scan#:3115) MassPeaks:186
RawMode:Averaged 18.565-18.575(3114-3116) BasePeak:101.10(7467)
BG Mode:Calc. from Peak Group 1 - Event 1 Scan

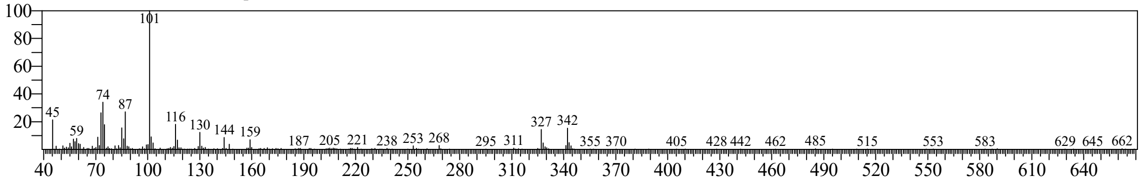

No hit compound

<< Target >>
Line#:6 R.Time:18.745(Scan#:3150) MassPeaks:143
RawMode:Averaged 18.740-18.750(3149-3151) BasePeak:87.05(2626)
BG Mode:Calc. from Peak Group 1 - Event 1 Scan

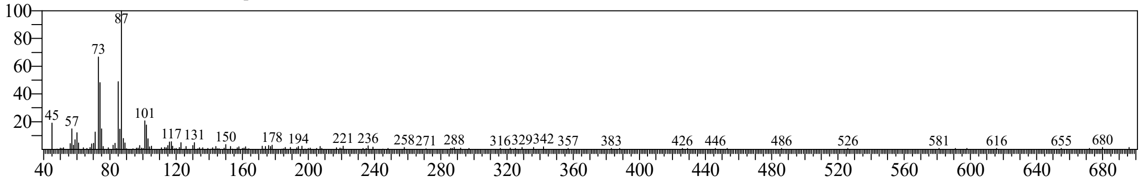

No hit compound

<< Target >>
Line#:7   R.Time:19.150(Scan#:3231) MassPeaks:114
RawMode:Averaged 19.145-19.155(3230-3232) BasePeak:73.05(21055)
BG Mode:Calc. from Peak Group 1 - Event 1 Scan

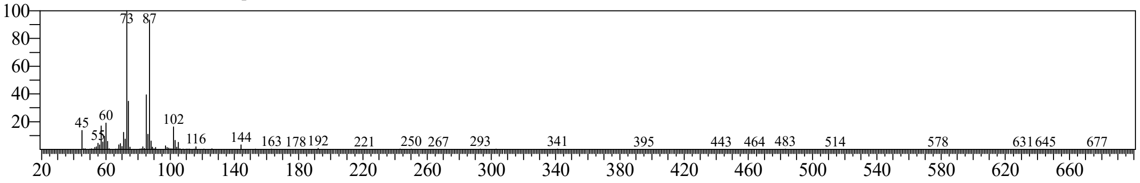

Hit#:1 Entry:64328 Library:NIST17.lib
SI:82 Formula:C7H14O6 CAS:0-00-0 MolWeight:194 RetIndex:1647
CompName:3-O-Methyl-d-glucose

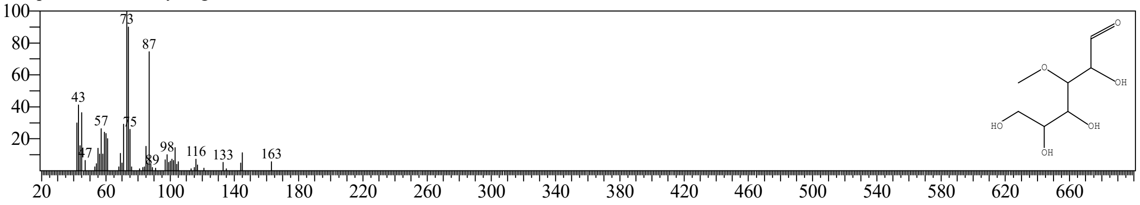

Hit#:2 Entry:64326 Library:NIST17.lib
SI:81 Formula:C7H14O6 CAS:4097-91-0 MolWeight:194 RetIndex:1667
CompName:.alpha.-Methyl mannofuranoside

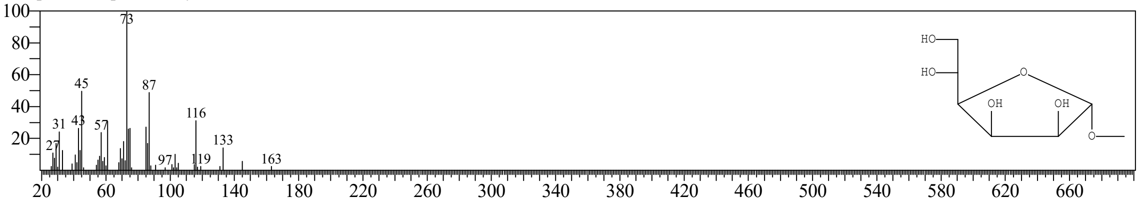

Hit#:3 Entry:64325 Library:NIST17.lib
SI:81 Formula:C7H14O6 CAS:0-00-0 MolWeight:194 RetIndex:1714
CompName:3-Methylmannoside

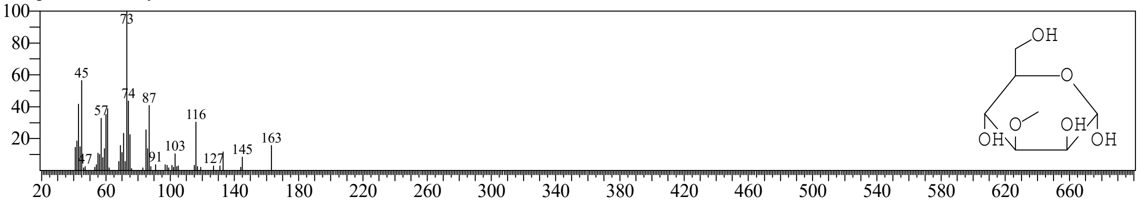

<< Target >>
Line#:8 R.Time:20.165(Scan#:3434) MassPeaks:176
RawMode:Averaged 20.160-20.170(3433-3435) BasePeak:192.05(12619)
BG Mode:Calc. from Peak Group 1 - Event 1 Scan

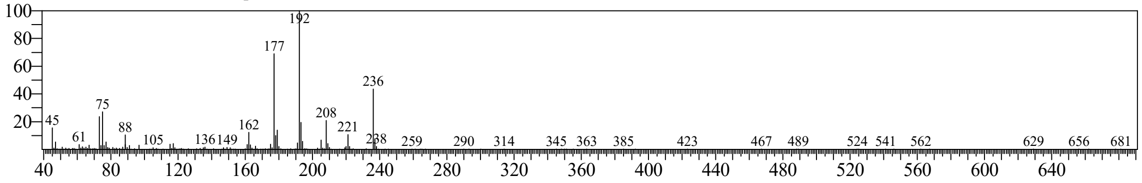

No hit compound

<< Target >>
Line#:9 R.Time:22.785(Scan#:3958) MassPeaks:172
RawMode:Averaged 22.780-22.790(3957-3959) BasePeak:73.05(2148)
BG Mode:Calc. from Peak Group 1 - Event 1 Scan

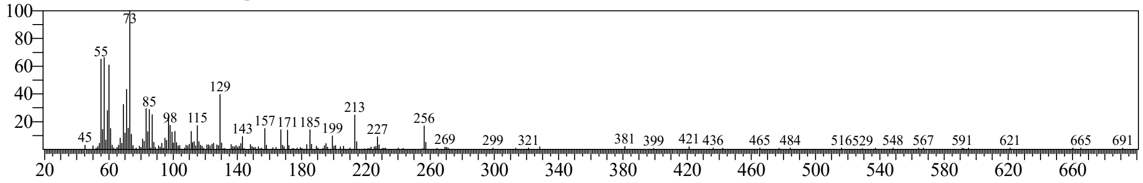

Hit#:1 Entry:129354 Library:NIST17.lib
SI:91 Formula:C16H32O2 CAS:57-10-3 MolWeight:256 RetIndex:1968
CompName:n-Hexadecanoic acid

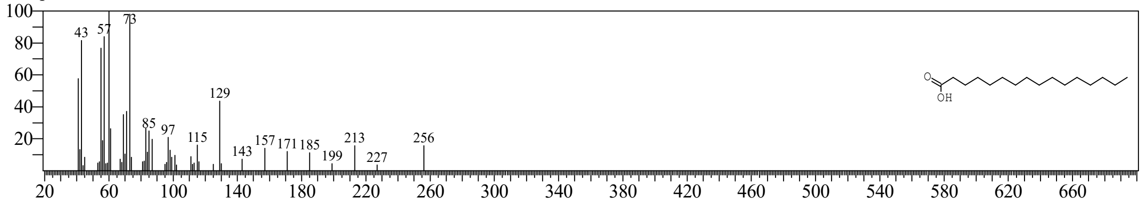

Hit#:2 Entry:114633 Library:NIST17.lib
SI:88 Formula:C15H30O2 CAS:1002-84-2 MolWeight:242 RetIndex:1869
CompName:Pentadecanoic acid

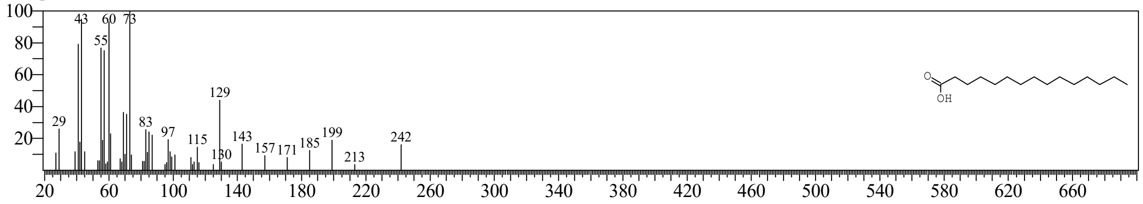

Hit#:3 Entry:190093 Library:NIST17.lib
SI:88 Formula:C20H40O2 CAS:506-30-9 MolWeight:312 RetIndex:2366
CompName:Eicosanoic acid

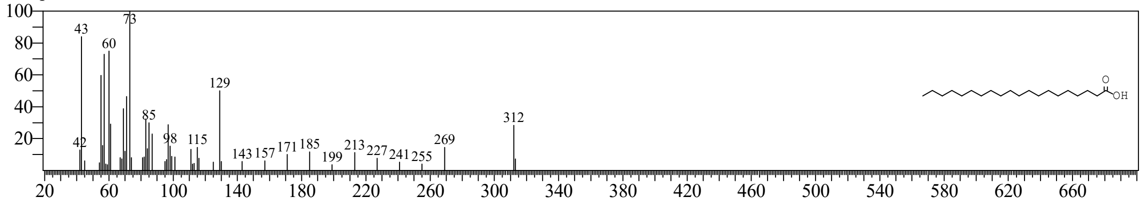

<< Target >>
Line#:10   R.Time:22.920(Scan#:3985) MassPeaks:153
RawMode:Averaged 22.915-22.925(3984-3986) BasePeak:59.00(17023)
BG Mode:Calc. from Peak Group 1 - Event 1 Scan

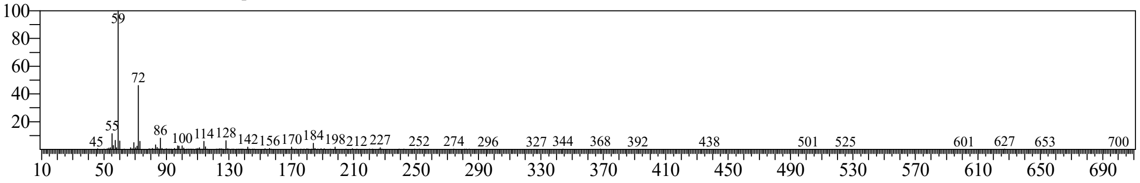

Hit#:1 Entry:99125 Library:NIST17.lib
SI:96 Formula:C14H29NO CAS:638-58-4 MolWeight:227 RetIndex:1822
CompName:Tetradecanamide

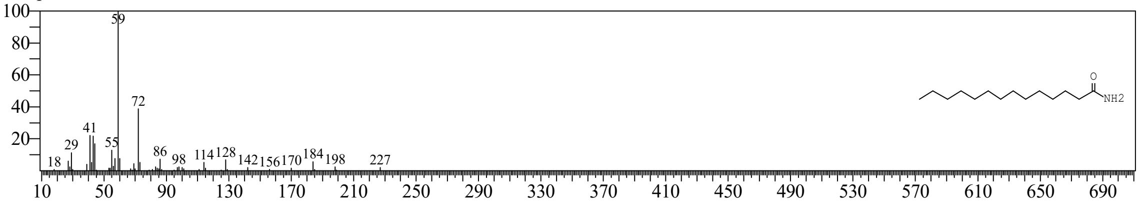

Hit#:2 Entry:128011 Library:NIST17.lib
SI:94 Formula:C16H33NO CAS:629-54-9 MolWeight:255 RetIndex:2021
CompName:Hexadecanamide

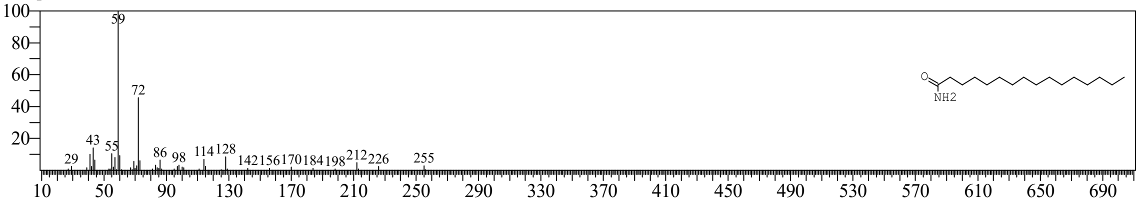

Hit#:3 Entry:158052 Library:NIST17.lib
SI:92 Formula:C18H37NO CAS:124-26-5 MolWeight:283 RetIndex:2220
CompName:Octadecanamide

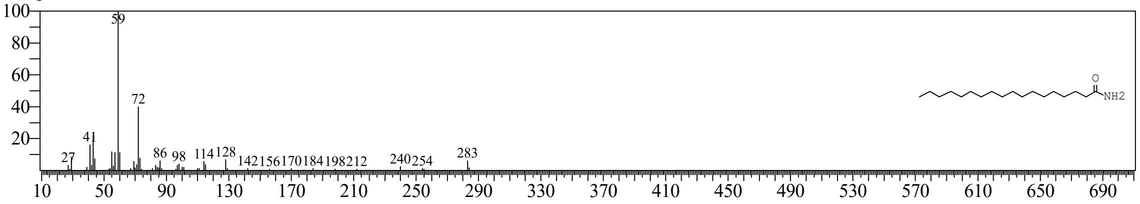

<< Target >>
Line#:11   R.Time:23.830(Scan#:4167) MassPeaks:198
RawMode:Averaged 23.825-23.835(4166-4168) BasePeak:117.05(4904)
BG Mode:Calc. from Peak Group 1 - Event 1 Scan

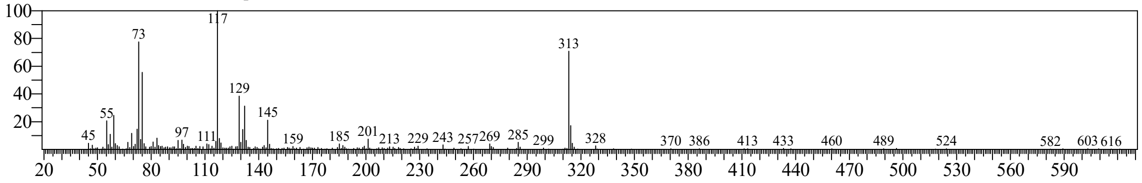

Hit#:1 Entry:206942 Library:NIST17.lib
SI:92 Formula:C19H40O2Si CAS:55520-89-3 MolWeight:328 RetIndex:1987
CompName:Palmitic Acid, TMS derivative

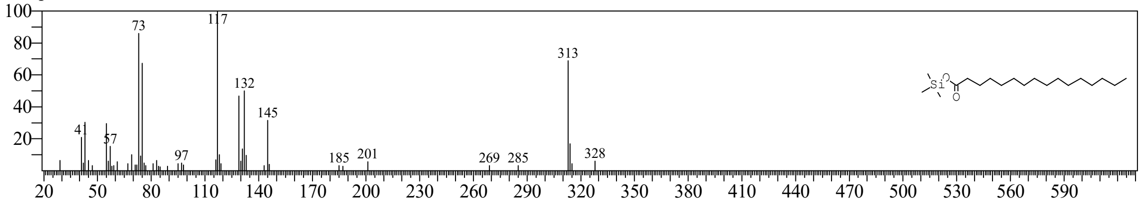

<< Target >>
Line#:12   R.Time:24.310(Scan#:4263) MassPeaks:218
RawMode:Averaged 24.305-24.315(4262-4264) BasePeak:73.05(3770)
BG Mode:Calc. from Peak Group 1 - Event 1 Scan

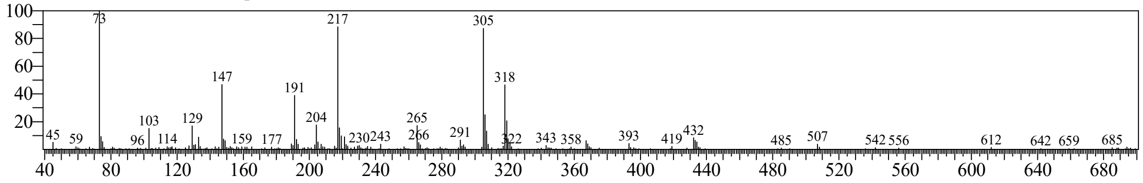

Hit#:1 Entry:303653 Library:NIST17.lib
SI:94 Formula:C24H60O6Si6 CAS:0-00-0 MolWeight:612 RetIndex:2194
CompName:1,2,3,4,5,6-Hexa-O-trimethelsilyl-myo-inositol

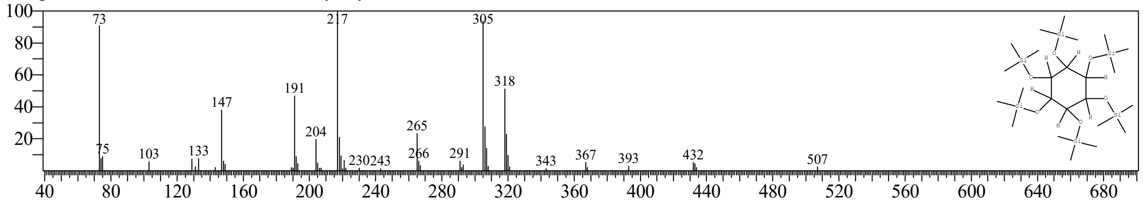

Hit#:2 Entry:303644 Library:NIST17.lib
SI:90 Formula:C24H60O6Si6 CAS:2582-79-8 MolWeight:612 RetIndex:2194
CompName:Myo-Inositol, 6TMS derivative

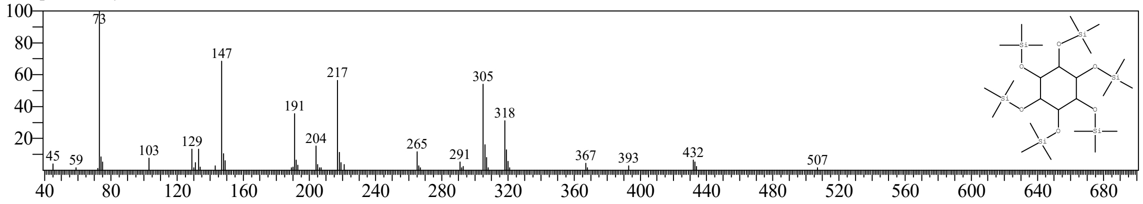

Hit#:3 Entry:303646 Library:NIST17.lib
SI:85 Formula:C24H60O6Si6 CAS:29267-01-4 MolWeight:612 RetIndex:2194
CompName:Inositol, epi-, 6TMS derivative

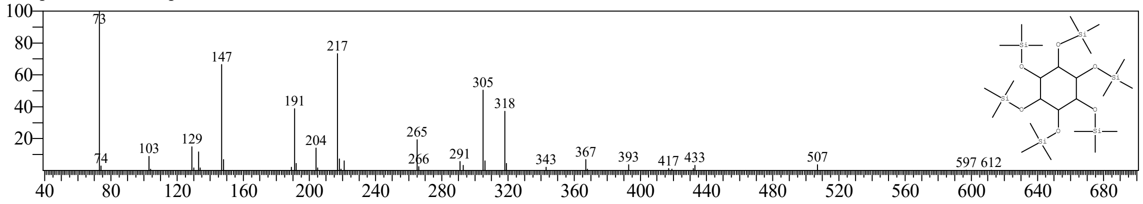

<< Target >>
Line#:13 R.Time:25.130(Scan#:4427) MassPeaks:195
RawMode:Averaged 25.125-25.135(4426-4428) BasePeak:59.00(26422)
BG Mode:Calc. from Peak Group 1 - Event 1 Scan

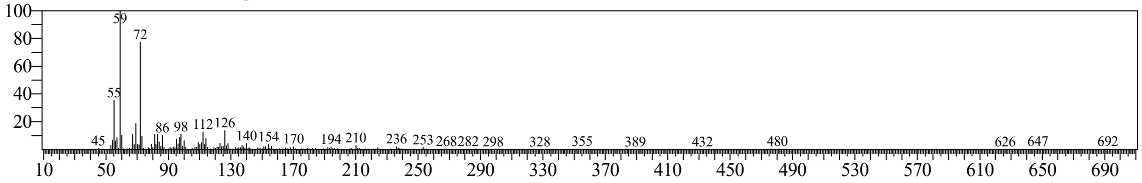

Hit#:1 Entry:125782 Library:NIST17.lib
SI:95 Formula:C16H31NO CAS:106010-22-4 MolWeight:253 RetIndex:2029
CompName:Palmitoleamide

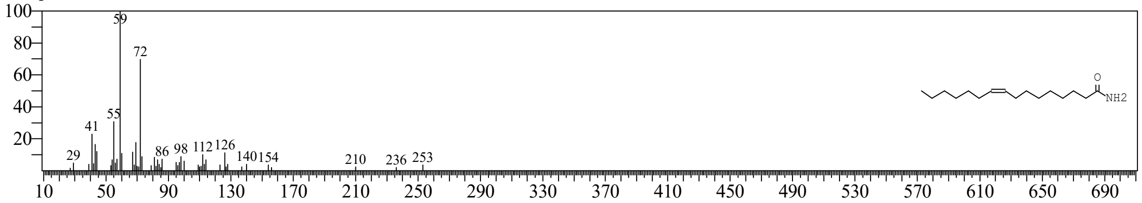

Hit#:2 Entry:216079 Library:NIST17.lib
SI:93 Formula:C22H43NO CAS:112-84-5 MolWeight:337 RetIndex:2625
CompName:13-Docosenamide, (Z)-

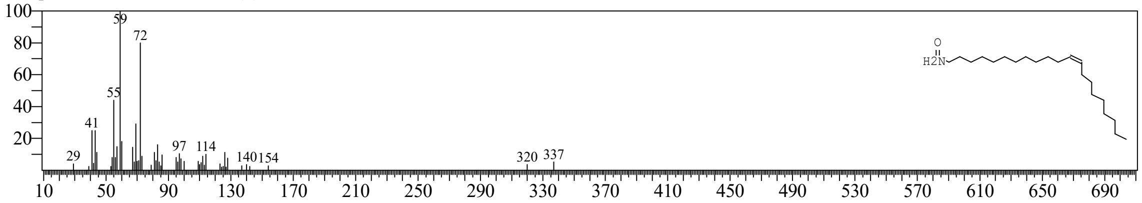

Hit#:3 Entry:155754 Library:NIST17.lib
SI:93 Formula:C18H35NO CAS:301-02-0 MolWeight:281 RetIndex:2228
CompName:9-Octadecenamide, (Z)-

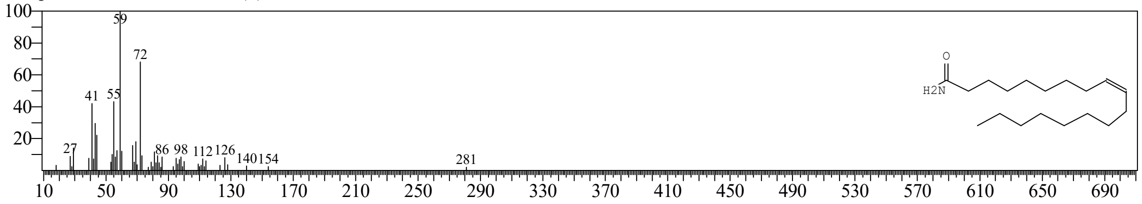

<< Target >>
Line#:14 R.Time:25.175(Scan#:4436) MassPeaks:173
RawMode:Averaged 25.170-25.180(4435-4437) BasePeak:59.05(2520)
BG Mode:Calc. from Peak Group 1 - Event 1 Scan

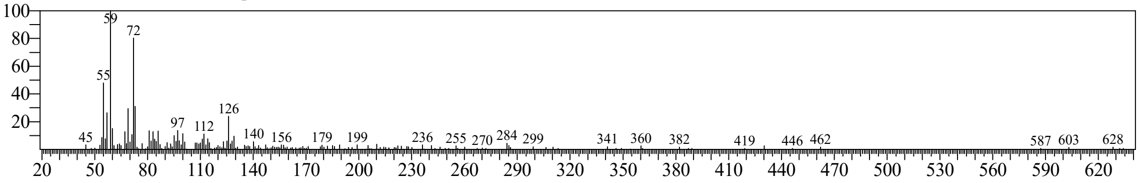

Hit#:1 Entry:216079 Library:NIST17.lib
SI:87 Formula:C22H43NO CAS:112-84-5 MolWeight:337 RetIndex:2625
CompName:13-Docosenamide, (Z)-

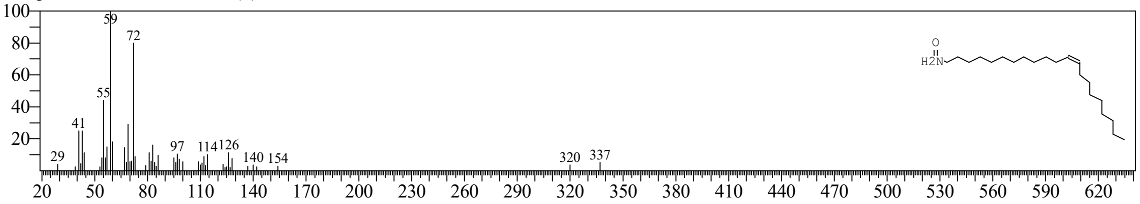

Hit#:2 Entry:155756 Library:NIST17.lib
SI:84 Formula:C18H35NO CAS:301-02-0 MolWeight:281 RetIndex:2228
CompName:9-Octadecenamide, (Z)-

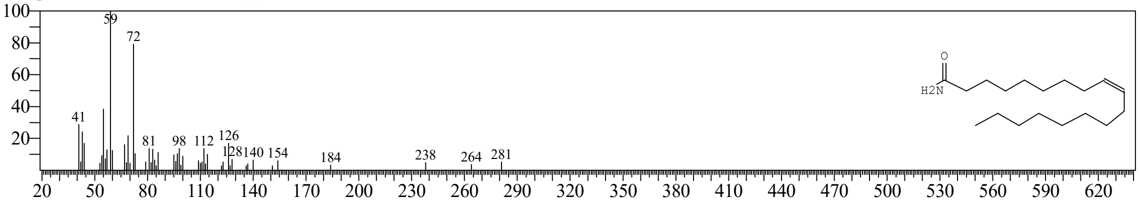

Hit#:3 Entry:125782 Library:NIST17.lib
SI:83 Formula:C16H31NO CAS:106010-22-4 MolWeight:253 RetIndex:2029
CompName:Palmitoleamide

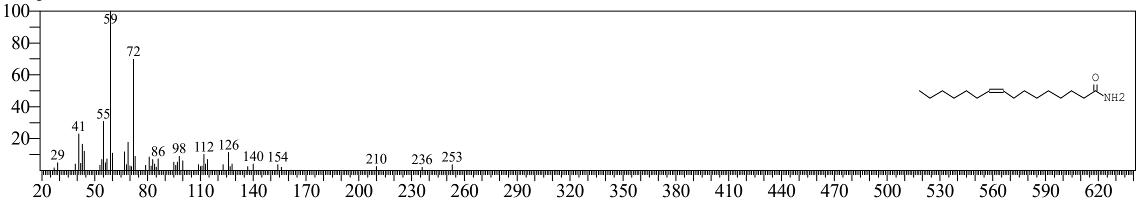

<< Target >>
Line#:15 R.Time:25.375(Scan#:4476) MassPeaks:229
RawMode:Averaged 25.370-25.380(4475-4477) BasePeak:59.05(853541)
BG Mode:Calc. from Peak Group 1 - Event 1 Scan

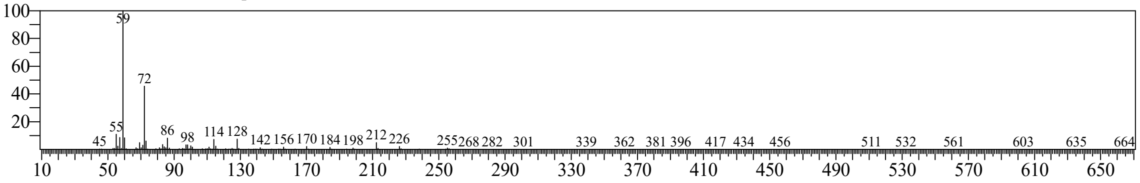

Hit#:1 Entry:128011 Library:NIST17.lib
SI:98 Formula:C16H33NO CAS:629-54-9 MolWeight:255 RetIndex:2021
CompName:Hexadecanamide

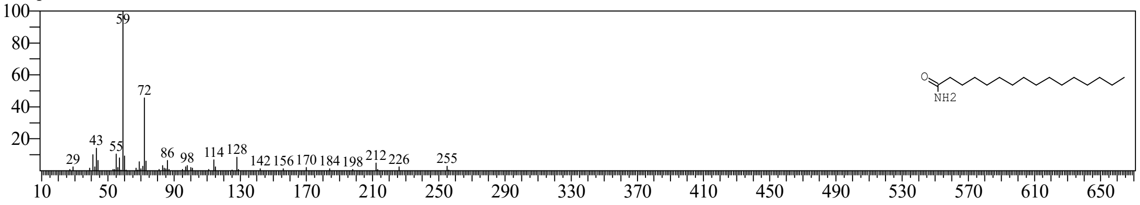

Hit#:2 Entry:99125 Library:NIST17.lib
SI:93 Formula:C14H29NO CAS:638-58-4 MolWeight:227 RetIndex:1822
CompName:Tetradecanamide

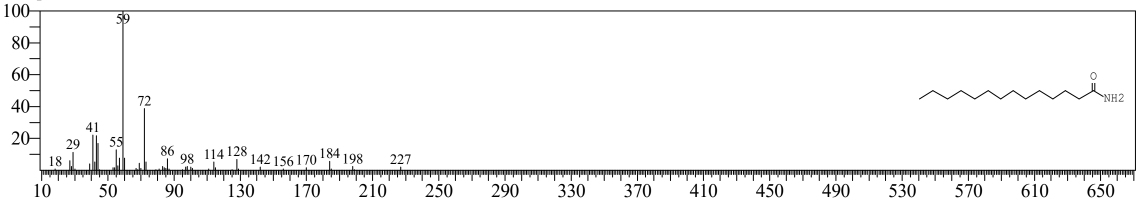

Hit#:3 Entry:158052 Library:NIST17.lib
SI:93 Formula:C18H37NO CAS:124-26-5 MolWeight:283 RetIndex:2220
CompName:Octadecanamide

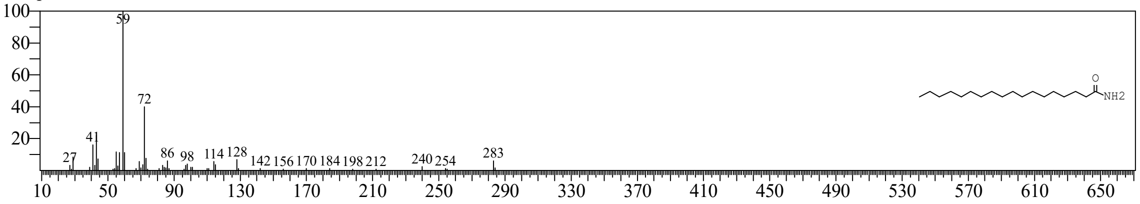

<< Target >>
Line#:16 R.Time:27.395(Scan#:4880) MassPeaks:297
RawMode:Averaged 27.390-27.400(4879-4881) BasePeak:59.05(783545)
BG Mode:Calc. from Peak Group 1 - Event 1 Scan

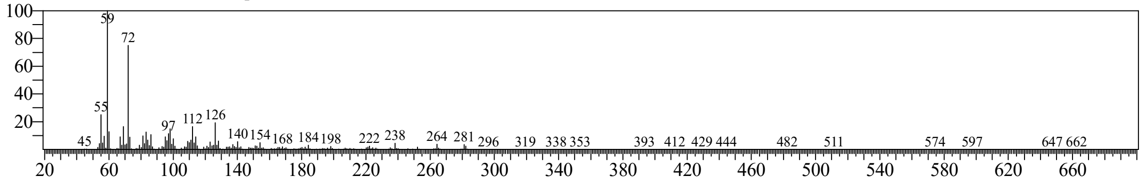

Hit#:1 Entry:155756 Library:NIST17.lib
SI:94 Formula:C18H35NO CAS:301-02-0 MolWeight:281 RetIndex:2228
CompName:9-Octadecenamide, (Z)-

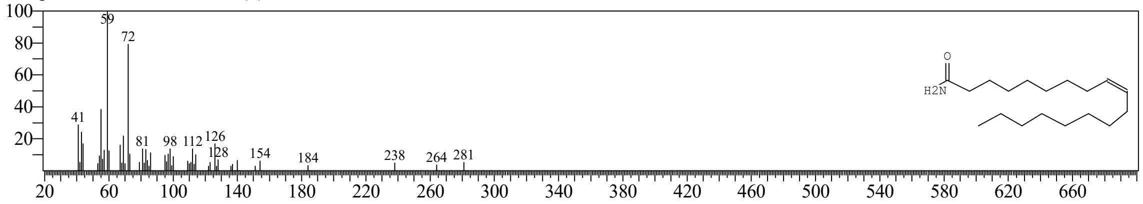

Hit#:2 Entry:216079 Library:NIST17.lib
SI:90 Formula:C22H43NO CAS:112-84-5 MolWeight:337 RetIndex:2625
CompName:13-Docosenamide, (Z)-

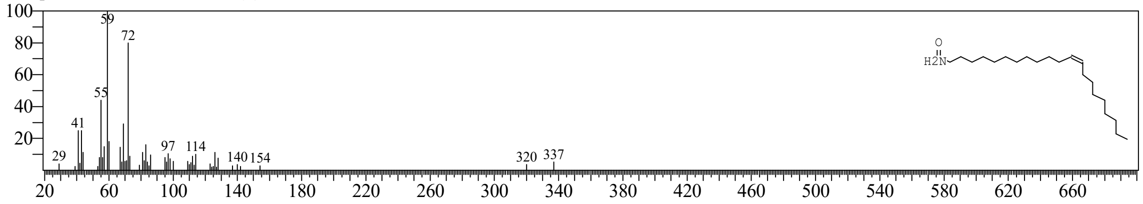

Hit#:3 Entry:125782 Library:NIST17.lib
SI:90 Formula:C16H31NO CAS:106010-22-4 MolWeight:253 RetIndex:2029
CompName:Palmitoleamide

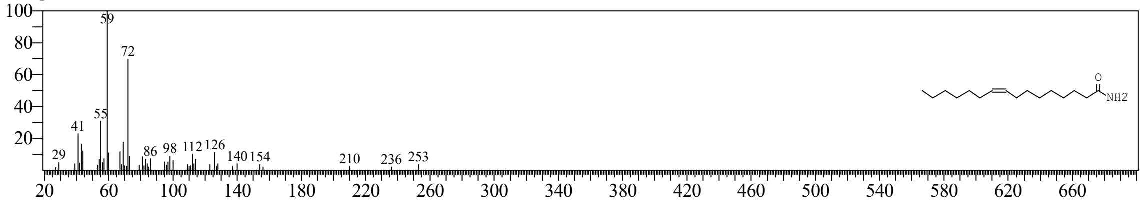

<< Target >>
Line#:17 R.Time:27.445(Scan#:4890) MassPeaks:239
RawMode:Averaged 27.440-27.450(4889-4891) BasePeak:59.05(334660)
BG Mode:Calc. from Peak Group 1 - Event 1 Scan

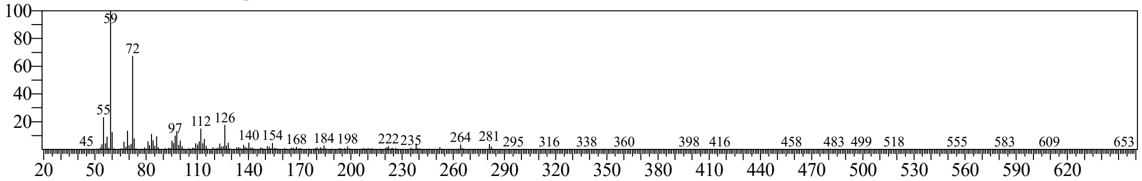

Hit#:1 Entry:155756 Library:NIST17.lib
SI:92 Formula:C18H35NO CAS:301-02-0 MolWeight:281 RetIndex:2228
CompName:9-Octadecenamide, (Z)-

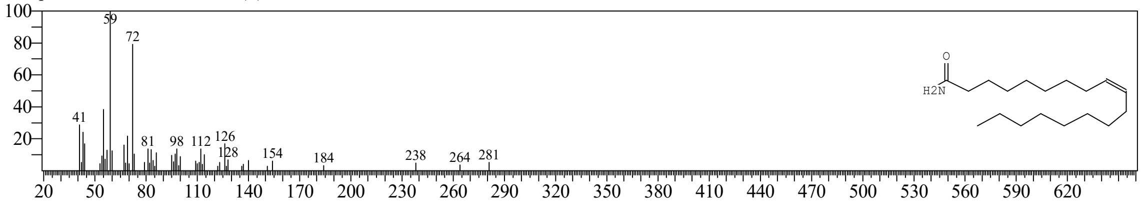

Hit#:2 Entry:125782 Library:NIST17.lib
SI:90 Formula:C16H31NO CAS:106010-22-4 MolWeight:253 RetIndex:2029
CompName:Palmitoleamide

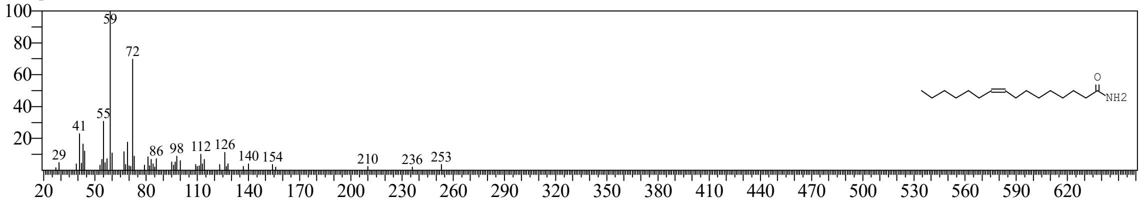

Hit#:3 Entry:216079 Library:NIST17.lib
SI:88 Formula:C22H43NO CAS:112-84-5 MolWeight:337 RetIndex:2625
CompName:13-Docosenamide, (Z)-

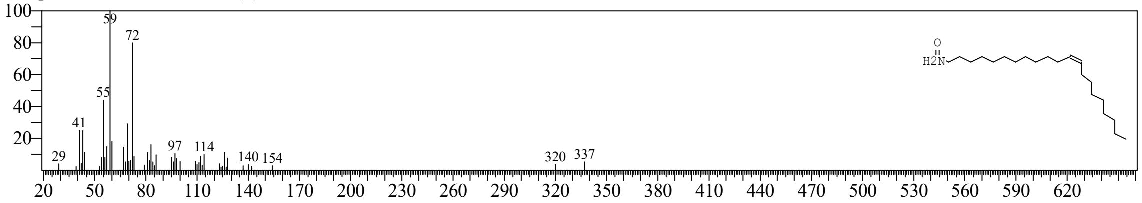

<< Target >>
Line#:18 R.Time:27.505(Scan#:4902) MassPeaks:274
RawMode:Averaged 27.500-27.510(4901-4903) BasePeak:59.05(552241)
BG Mode:Calc. from Peak Group 1 - Event 1 Scan

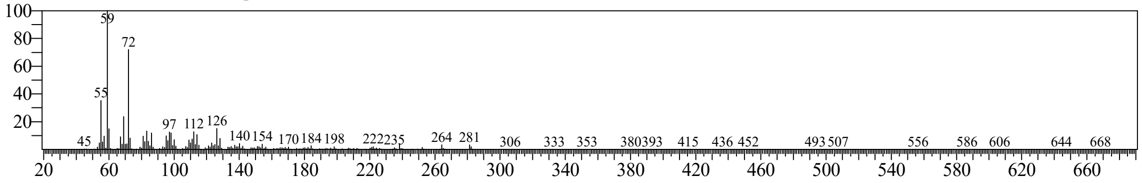

Hit#:1 Entry:155756 Library:NIST17.lib
SI:94 Formula:C18H35NO CAS:301-02-0 MolWeight:281 RetIndex:2228
CompName:9-Octadecenamide, (Z)-

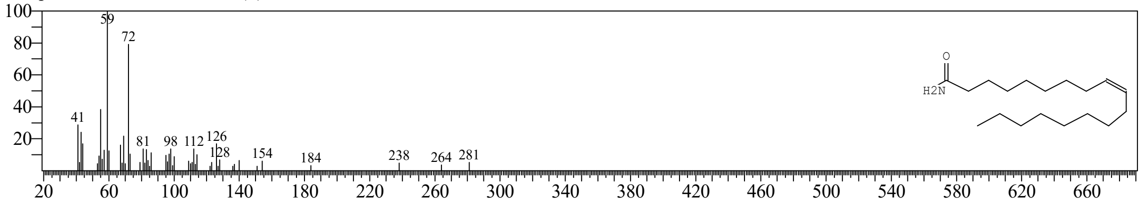

Hit#:2 Entry:216079 Library:NIST17.lib
SI:92 Formula:C22H43NO CAS:112-84-5 MolWeight:337 RetIndex:2625
CompName:13-Docosenamide, (Z)-

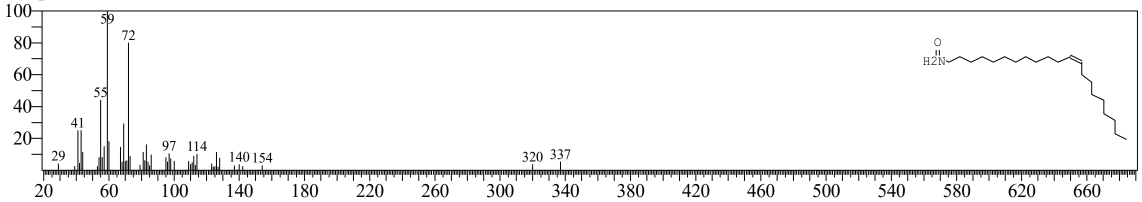

Hit#:3 Entry:125782 Library:NIST17.lib
SI:90 Formula:C16H31NO CAS:106010-22-4 MolWeight:253 RetIndex:2029
CompName:Palmitoleamide

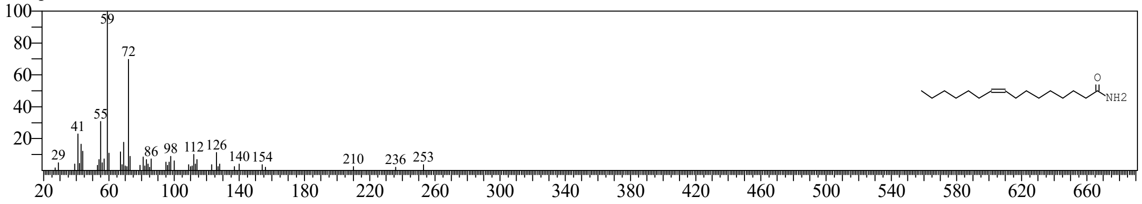

<< Target >>
Line#:19 R.Time:27.625(Scan#:4926) MassPeaks:268
RawMode:Averaged 27.620-27.630(4925-4927) BasePeak:59.05(600608)
BG Mode:Calc. from Peak Group 1 - Event 1 Scan

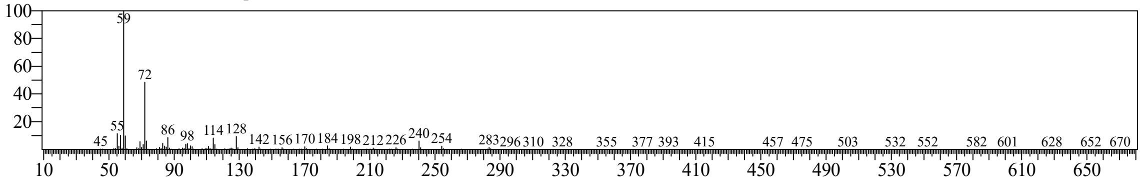

Hit#:1 Entry:158052 Library:NIST17.lib
SI:95 Formula:C18H37NO CAS:124-26-5 MolWeight:283 RetIndex:2220
CompName:Octadecanamide

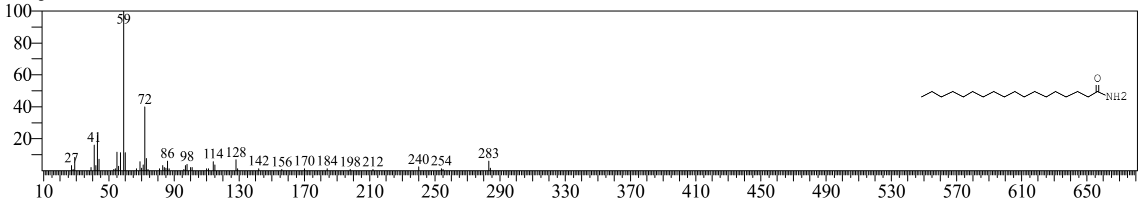

Hit#:2 Entry:128011 Library:NIST17.lib
SI:94 Formula:C16H33NO CAS:629-54-9 MolWeight:255 RetIndex:2021
CompName:Hexadecanamide

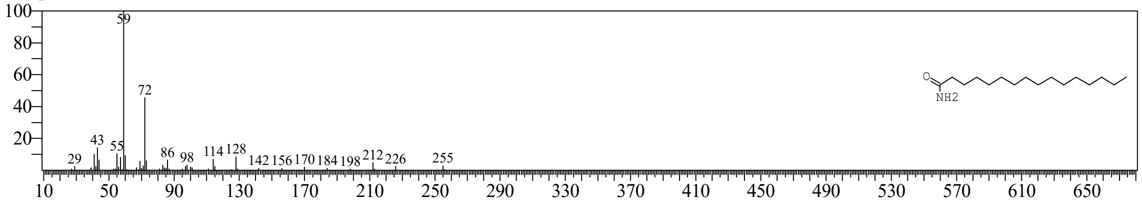

Hit#:3 Entry:99125 Library:NIST17.lib
SI:91 Formula:C14H29NO CAS:638-58-4 MolWeight:227 RetIndex:1822
CompName:Tetradecanamide

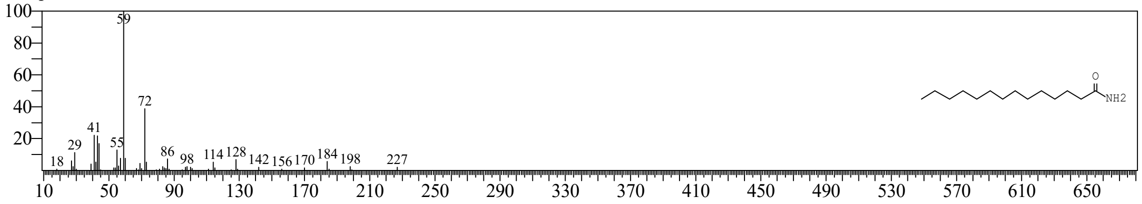

<< Target >>
Line#:20 R.Time:27.830(Scan#:4967) MassPeaks:250
RawMode:Averaged 27.825-27.835(4966-4968) BasePeak:59.05(5904)
BG Mode:Calc. from Peak Group 1 - Event 1 Scan

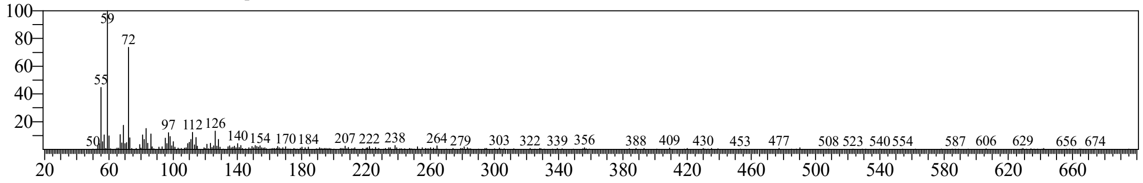

Hit#:1 Entry:216079 Library:NIST17.lib
SI:92 Formula:C22H43NO CAS:112-84-5 MolWeight:337 RetIndex:2625
CompName:13-Docosenamide, (Z)-

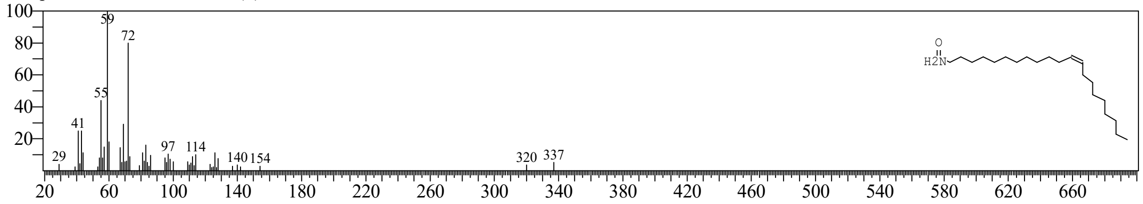

Hit#:2 Entry:155756 Library:NIST17.lib
SI:92 Formula:C18H35NO CAS:301-02-0 MolWeight:281 RetIndex:2228
CompName:9-Octadecenamide, (Z)-

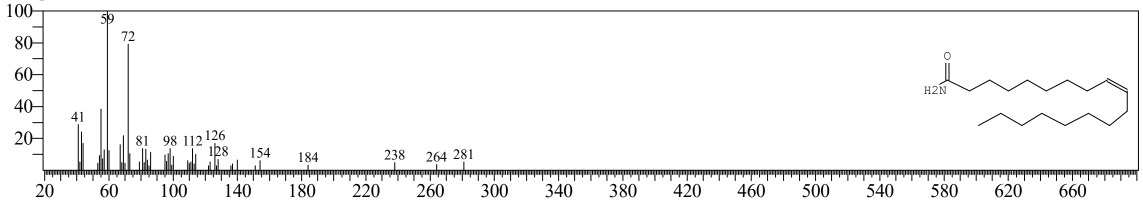

Hit#:3 Entry:125782 Library:NIST17.lib
SI:90 Formula:C16H31NO CAS:106010-22-4 MolWeight:253 RetIndex:2029
CompName:Palmitoleamide

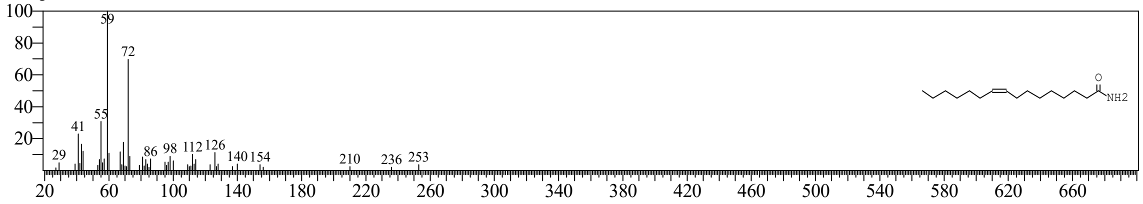

<< Target >>
Line#:21 R.Time:29.430(Scan#:5287) MassPeaks:284
RawMode:Averaged 29.425-29.435(5286-5288) BasePeak:59.05(286616)
BG Mode:Calc. from Peak Group 1 - Event 1 Scan

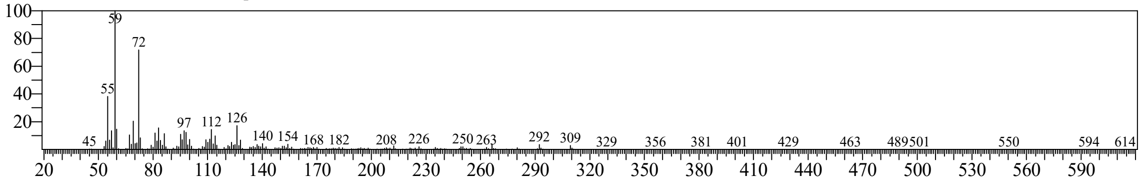

Hit#:1 Entry:155756 Library:NIST17.lib
SI:93 Formula:C18H35NO CAS:301-02-0 MolWeight:281 RetIndex:2228
CompName:9-Octadecenamide, (Z)-

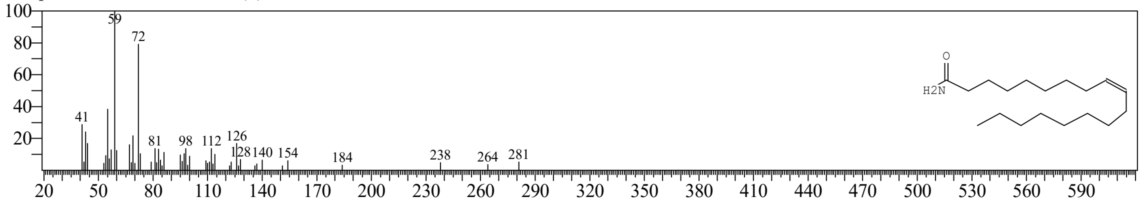

Hit#:2 Entry:216079 Library:NIST17.lib
SI:92 Formula:C22H43NO CAS:112-84-5 MolWeight:337 RetIndex:2625
CompName:13-Docosenamide, (Z)-

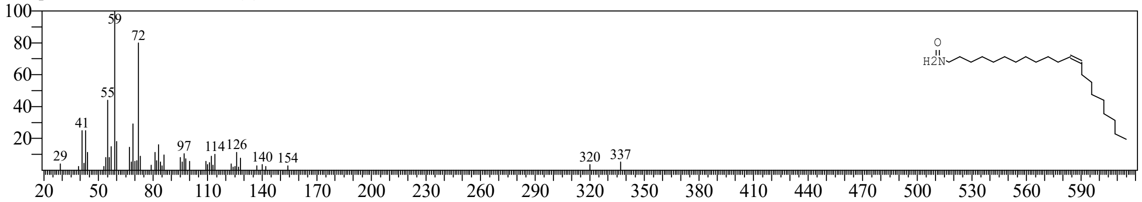

Hit#:3 Entry:125782 Library:NIST17.lib
SI:90 Formula:C16H31NO CAS:106010-22-4 MolWeight:253 RetIndex:2029
CompName:Palmitoleamide

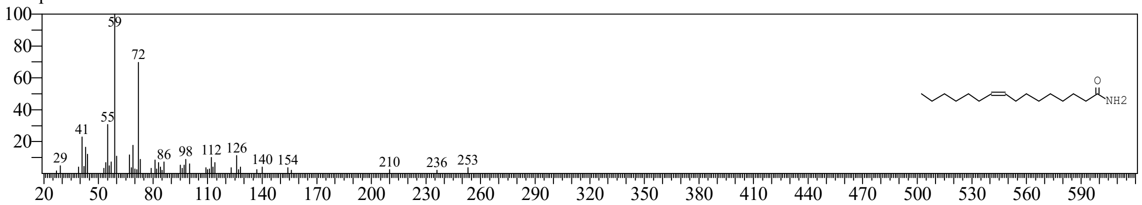

<< Target >>
Line#:22 R.Time:29.490(Scan#:5299) MassPeaks:276
RawMode:Averaged 29.485-29.495(5298-5300) BasePeak:59.05(100734)
BG Mode:Calc. from Peak Group 1 - Event 1 Scan

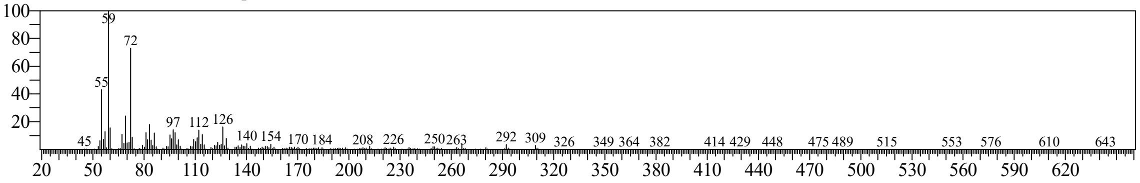

Hit#:1 Entry:216079 Library:NIST17.lib
SI:93 Formula:C22H43NO CAS:112-84-5 MolWeight:337 RetIndex:2625
CompName:13-Docosenamide, (Z)-

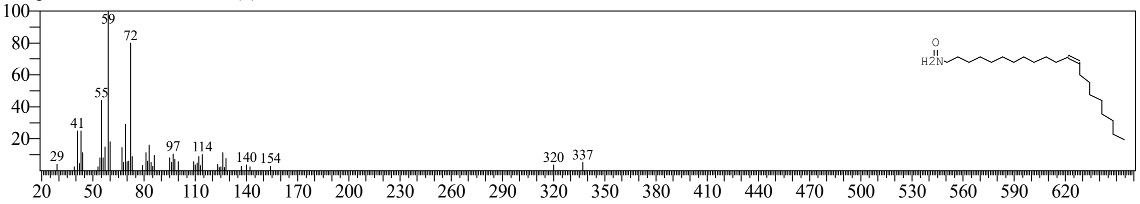

Hit#:2 Entry:155756 Library:NIST17.lib
SI:92 Formula:C18H35NO CAS:301-02-0 MolWeight:281 RetIndex:2228
CompName:9-Octadecenamide, (Z)-

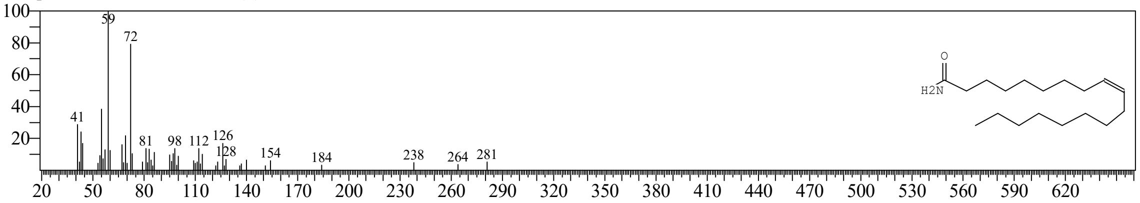

Hit#:3 Entry:125782 Library:NIST17.lib
SI:89 Formula:C16H31NO CAS:106010-22-4 MolWeight:253 RetIndex:2029
CompName:Palmitoleamide

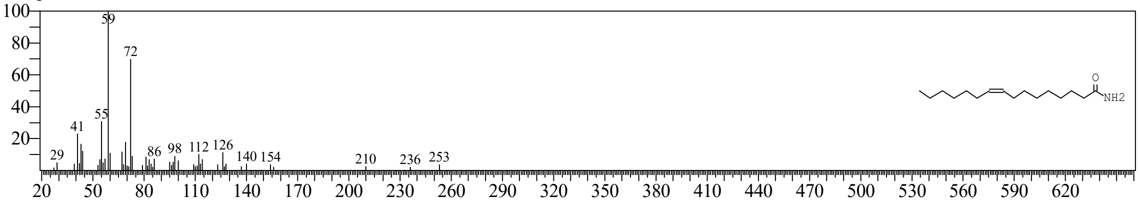

<< Target >>
Line#:23 R.Time:29.660(Scan#:5333) MassPeaks:304
RawMode:Averaged 29.655-29.665(5332-5334) BasePeak:59.05(53825)
BG Mode:Calc. from Peak Group 1 - Event 1 Scan

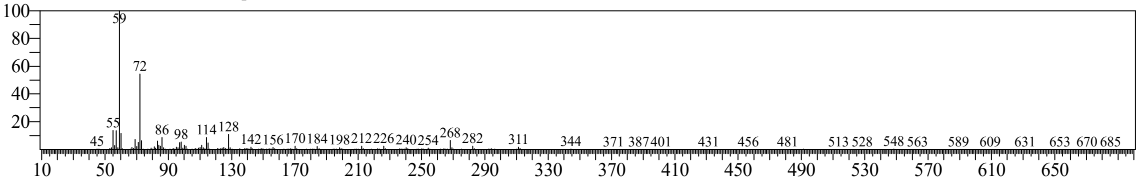

Hit#:1 Entry:128011 Library:NIST17.lib
SI:91 Formula:C16H33NO CAS:629-54-9 MolWeight:255 RetIndex:2021
CompName:Hexadecanamide

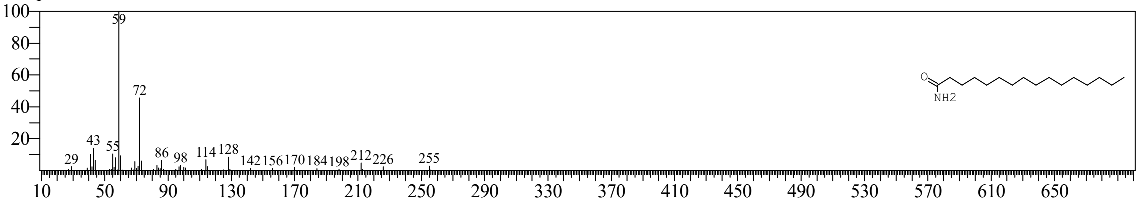

Hit#:2 Entry:158052 Library:NIST17.lib
SI:90 Formula:C18H37NO CAS:124-26-5 MolWeight:283 RetIndex:2220
CompName:Octadecanamide

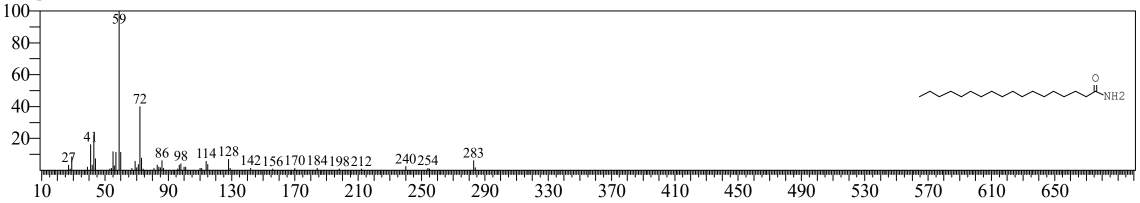

Hit#:3 Entry:99164 Library:NIST17.lib
SI:88 Formula:C14H29NO CAS:638-58-4 MolWeight:227 RetIndex:1822
CompName:Tetradecanamide

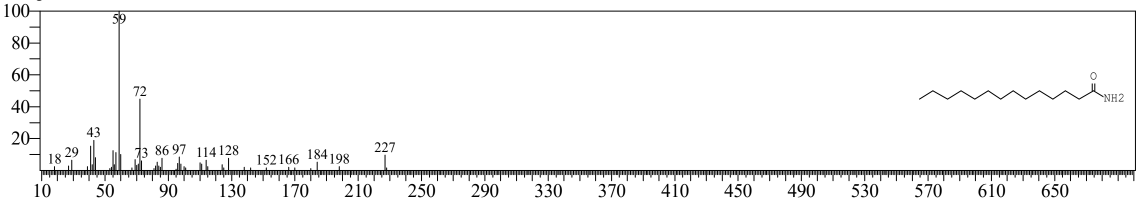

<< Target >>
Line#:24 R.Time:31.430(Scan#:5687) MassPeaks:260
RawMode:Averaged 31.425-31.435(5686-5688) BasePeak:59.00(2673)
BG Mode:Calc. from Peak Group 1 - Event 1 Scan

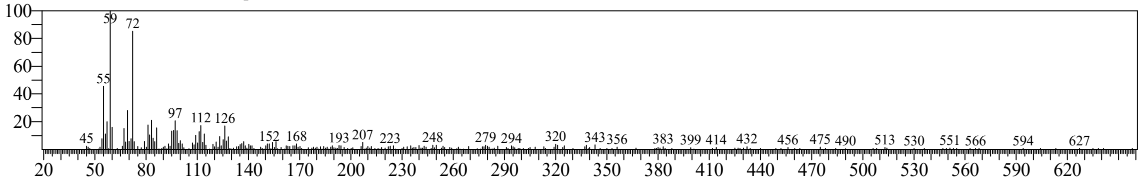

Hit#:1 Entry:216079 Library:NIST17.lib
SI:89 Formula:C22H43NO CAS:112-84-5 MolWeight:337 RetIndex:2625
CompName:13-Docosenamide, (Z)-

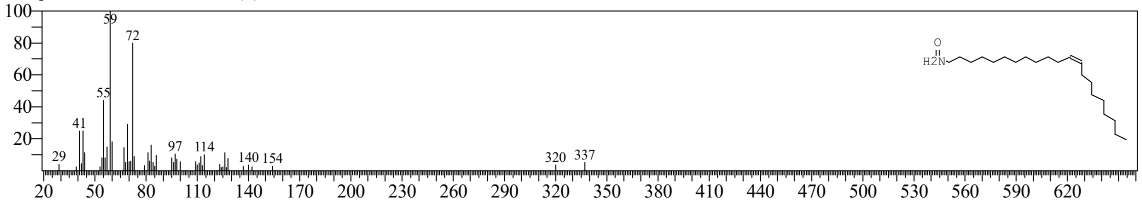

Hit#:2 Entry:155756 Library:NIST17.lib
SI:88 Formula:C18H35NO CAS:301-02-0 MolWeight:281 RetIndex:2228
CompName:9-Octadecenamide, (Z)-

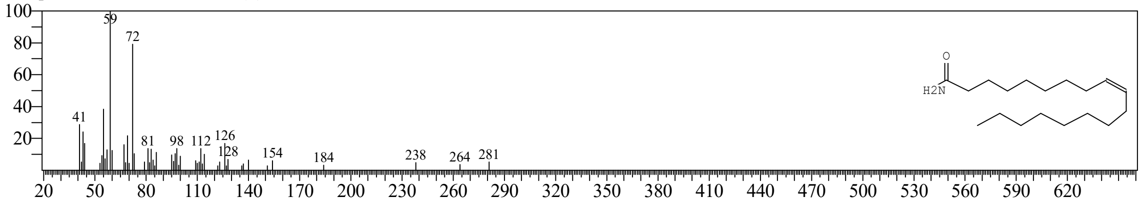

Hit#:3 Entry:125782 Library:NIST17.lib
SI:83 Formula:C16H31NO CAS:106010-22-4 MolWeight:253 RetIndex:2029
CompName:Palmitoleamide

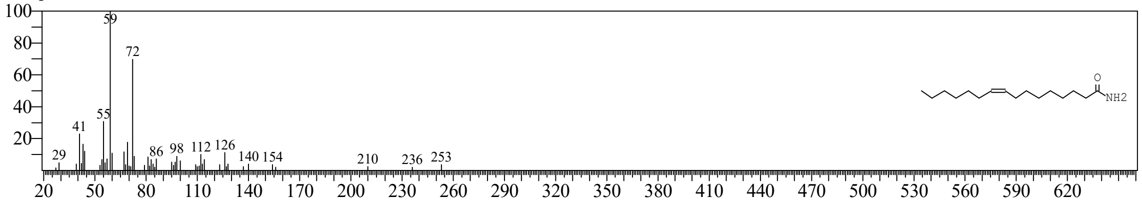

<< Target >>
Line#:25 R.Time:33.955(Scan#:6192) MassPeaks:369
RawMode:Averaged 33.950-33.960(6191-6193) BasePeak:239.15(150561)
BG Mode:Calc. from Peak Group 1 - Event 1 Scan

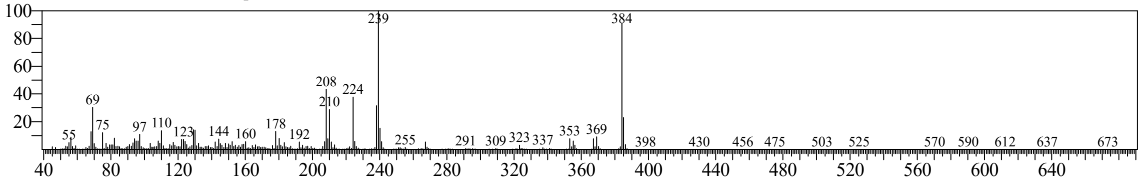

No hit compound

<< Target >>
Line#:26   R.Time:35.825(Scan#:6566) MassPeaks:336
RawMode:Averaged 35.820-35.830(6565-6567) BasePeak:223.10(17013)
BG Mode:Calc. from Peak Group 1 - Event 1 Scan

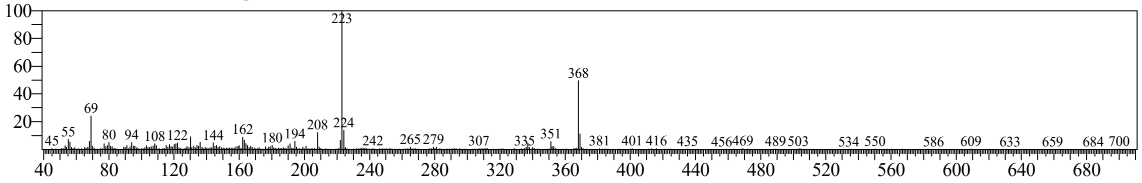

Hit#:1 Entry:243500 Library:NIST17.lib
SI:84 Formula:C21H24N2O4 CAS:509-80-8 MolWeight:368 RetIndex:2808
CompName:Formosanan-16-carboxylic acid, 19-methyl-2-oxo-, methyl ester, (19.alpha.)-

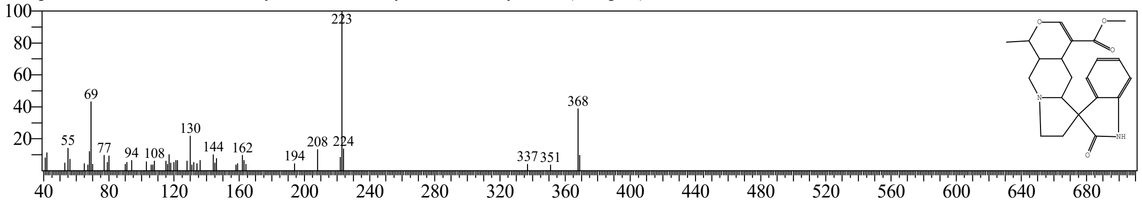

Peak Report TIC

Peak#	R.Time	I.Time	F.Time	Area	Area%	Height	Height%	A/H	Name
1	16.275	16.220	16.305	17152	0.02	7995	0.03	2.15	.beta.-D-Glucopyranose, 1,6-anhydro-
2	17.248	17.205	17.320	138627	0.14	48680	0.16	2.85	
3	18.030	17.975	18.050	121552	0.13	44446	0.15	2.73	
4	18.095	18.050	18.195	497722	0.52	110821	0.36	4.49	
5	18.569	18.535	18.595	75158	0.08	37342	0.12	2.01	
6	18.746	18.710	18.775	29430	0.03	12602	0.04	2.34	
7	19.149	19.060	19.200	614617	0.64	105470	0.35	5.83	3-O-Methyl-d-glucose
8	20.165	20.130	20.220	127964	0.13	69410	0.23	1.84	
9	22.783	22.755	22.815	44924	0.05	24181	0.08	1.86	n-Hexadecanoic acid
10	22.920	22.825	22.975	101178	0.11	48578	0.16	2.08	Tetradecanamide
11	24.312	24.285	24.350	59325	0.06	34438	0.11	1.72	1,2,3,4,5,6-Hexa-O-trimethelsilyl-myo-inositol
12	25.131	25.090	25.160	332342	0.35	173275	0.57	1.92	Palmitoleamide
13	25.176	25.160	25.225	117027	0.12	53270	0.18	2.20	13-Docosenamide, (Z)-
14	25.376	25.320	25.480	4765506	4.95	2436008	8.02	1.96	Hexadecanamide
15	27.393	27.195	27.420	34626667	35.98	8181290	26.93	4.23	9-Octadecenamide, (Z)-
16	27.445	27.420	27.470	14047995	14.60	5481981	18.05	2.56	9-Octadecenamide, (Z)-
17	27.504	27.470	27.585	18181665	18.89	6106336	20.10	2.98	9-Octadecenamide, (Z)-
18	27.624	27.585	27.770	5581438	5.80	2119088	6.98	2.63	Octadecanamide
19	27.831	27.770	27.875	151414	0.16	46007	0.15	3.29	13-Docosenamide, (Z)-
20	29.428	29.345	29.460	5077341	5.28	2302710	7.58	2.20	9-Octadecenamide, (Z)-
21	29.489	29.460	29.620	3400972	3.53	1121161	3.69	3.03	13-Docosenamide, (Z)-
22	29.660	29.620	29.780	555305	0.58	221304	0.73	2.51	Hexadecanamide
23	31.429	31.400	31.465	45299	0.05	23265	0.08	1.95	13-Docosenamide, (Z)-
24	33.954	33.825	34.135	6662714	6.92	1473133	4.85	4.52	
25	35.824	35.695	36.015	855074	0.89	92368	0.30	9.26	Formosanan-16-carboxylic acid, 19-methyl-2-oxo-, methyl ester, (19.alpha.)-
				96228408	100.00	30375159	100.00		

Method

[Comment]

===== Analytical Line 1 =====

[AOC-20i+s]
# of Rinses with Presolvent :2
# of Rinses with Solvent(post) :3
# of Rinses with Sample :3
Plunger Speed(Suction) :High
Viscosity Comp. Time :0.5 sec
Plunger Speed(Injection) :High
Syringe Insertion Speed :High
Injection Mode :Normal
Pumping Times :5
Inj. Port Dwell Time :0.3 sec
Terminal Air Gap :No
Plunger Washing Speed :High
Washing Volume :8uL
Syringe Suction Position :-2.0 mm
Syringe Injection Position :0.0 mm
Solvent Selection :only C
Column Oven Temp. :60.0 ?C
Injection Temp. :250.00 ?C

Injection Mode	:Split
Flow Control Mode	:Column Flow
Pressure	:8.327 psi
Total Flow	:24.0 mL/min
Column Flow	:1.00 mL/min
Linear Velocity	:36.5 cm/sec
Purge Flow	:3.0 mL/min
Split Ratio	:20.0
High Pressure Injection	:OFF
Carrier Gas Saver	:OFF
Splitter Hold	:OFF

Oven Temp. Program

Rate	Temperature(?C)	Hold Time(min)
-	60.0	3.00
8.00	280.0	25.00

Oven Cooling Rate :Middle

< Ready Check Heat Unit >

Column Oven	: Yes
SPL1	: Yes
MS	: Yes

< Ready Check Detector(FTD/BID) >

< Ready Check Baseline Drift >

< Ready Check Injection Flow >

SPL1 Carrier	: Yes
SPL1 Purge	: Yes

< Ready Check APC Flow >

< Ready Check Detector APC Flow >

External Wait	:No
Equilibrium Time	:2.0 min
Auto-flame On	:OFF
Auto-flame Off	:ON
Reignite	:OFF
Auto-zero after Ready	:ON
PrepRun Start	:Auto

[GC Program]

[GCMS-QP2020 NX]

IonSourceTemp	:250.00 ?C
Interface Temp.	:250.00 ?C
Solvent Cut Time	:2.50 min
Detector Gain Mode	:Relative to the Tuning Result
Detector Gain	:0.78 kV +0.00 kV
Threshold	:50

[MS Table]

--Group 1 - Event 1--

Start Time	:3.00min
End Time	:55.50min
ACQ Mode	:Scan
Event Time	:0.30sec
Scan Speed	:2500
Start m/z	:45.00
End m/z	:700.00

Sample Inlet Unit :GC

[MS Program]
Use MS Program :OFF
